# PCSK9 deficiency alters brain lipid composition without affecting brain development and function

**DOI:** 10.3389/fnmol.2022.1084633

**Published:** 2023-01-17

**Authors:** Angela Pärn, Ditte Olsen, Jürgen Tuvikene, Mathias Kaas, Ekaterina Borisova, Mesut Bilgin, Mie Elhauge, Joachim Vilstrup, Peder Madsen, Mateusz C. Ambrozkiewicz, Roman U. Goz, Tõnis Timmusk, Victor Tarabykin, Camilla Gustafsen, Simon Glerup

**Affiliations:** ^1^Department of Biomedicine, Aarhus University, Aarhus, Denmark; ^2^Department of Chemistry and Biotechnology, Tallinn University of Technology, Tallinn, Estonia; ^3^Protobios LLC, Tallinn, Estonia; ^4^Institute of Cell Biology and Neurobiology, Charité - Universitätsmedizin Berlin, Berlin, Germany; ^5^Tomsk National Research Medical Center of the Russian Academy of Sciences, Research Institute of Medical Genetics, Tomsk, Russia; ^6^Danish Cancer Society Research Center, Lipidomics Core Facility, Copenhagen, Denmark; ^7^Draupnir Bio ApS, INCUBA Skejby, Aarhus, Denmark; ^8^Department of Neurobiology, University of Pittsburgh Medical School, Pittsburgh, PA, United States

**Keywords:** proprotein convertase subtilisin/kexin type 9, cholesterol, lipidomics, LDLR, mouse behavior, brain lipids, brain functioning, brain development

## Abstract

PCSK9 induces lysosomal degradation of the low-density lipoprotein (LDL) receptor (LDLR) in the liver, hereby preventing removal of LDL cholesterol from the circulation. Accordingly, PCSK9 inhibitory antibodies and siRNA potently reduce LDL cholesterol to unprecedented low levels and are approved for treatment of hypercholesterolemia. In addition, PCSK9 inactivation alters the levels of several other circulating lipid classes and species. Brain function is critically influenced by cholesterol and lipid composition. However, it remains unclear how the brain is affected long-term by the reduction in circulating lipids as achieved with potent lipid lowering therapeutics such as PCSK9 inhibitors. Furthermore, it is unknown if locally expressed PCSK9 affects neuronal circuits through regulation of receptor levels. We have studied the effect of lifelong low peripheral cholesterol levels on brain lipid composition and behavior in adult PCSK9 KO mice. In addition, we studied the effect of PCSK9 on neurons in culture and *in vivo* in the developing cerebral cortex. We found that PCSK9 reduced LDLR and neurite complexity in cultured neurons, but neither PCSK9 KO nor overexpression affected cortical development *in vivo*. Interestingly, PCSK9 deficiency resulted in changes of several lipid classes in the adult cortex and cerebellum. Despite the observed changes, PCSK9 KO mice had unchanged behavior compared to WT controls. In conclusion, our findings demonstrate that altered PCSK9 levels do not compromise brain development or function in mice, and are in line with clinical trials showing that PCSK9 inhibitors have no adverse effects on cognitive function.

## 1. Introduction

Proprotein convertase subtilisin/kexin type 9 (PCSK9) was discovered in 2003 and initially named neural apoptosis-regulated convertase-1 (NARC-1; Seidah et al., 2003). Carriers of PCSK9 loss-of-function (LOF) mutations have low LDL cholesterol and reduced risk of adverse cardiovascular events (Cohen et al., 2005, 2006; Berge et al., 2006). PCSK9 binds the LDL receptor (LDLR) on the surface of hepatocytes and destines it for lysosomal degradation, resulting in decreased uptake of LDL particles by the liver (Maxwell et al., 2005; Lagace et al., 2006; Zhang et al., 2007; Poirier et al., 2009). Accordingly, PCSK9 siRNA and monoclonal antibodies blocking the PCSK9/LDLR interaction potently lower LDL cholesterol and reduce the risk of adverse cardiovascular events (Sabatine et al., 2017; Schwartz et al., 2018; Ray et al., 2020). The liver has the highest level of PCSK9 expression and is the main source of plasma PCSK9 (Seidah et al., 2003; Lagace et al., 2006). PCSK9 is also expressed by several other tissues, including the kidney, lung, intestine and brain (Seidah et al., 2003) but the role of extrahepatic PCSK9 remains unclear. In the brain, the expression peaks during cortical and cerebellar development, however, PCSK9 expression is also observed in adult mouse and human cerebellum (Seidah et al., 2003; gtexportal.org) and can be measured in human cerebrospinal fluid (CSF) (Persson et al., 2010; Chen et al., 2014). The regulation of PCSK9 in plasma and CSF appears to be different and, unlike in plasma, PCSK9 levels in CSF are not affected by diurnal rhythm (Chen et al., 2014). The role of PCSK9 in the brain still remains unclear but previous studies suggest that it may be involved in neuronal differentiation and apoptosis (Rashid et al., 2005; Poirier et al., 2006), and influence cholesterol metabolism in cultured neuronal cells (Papotti et al., 2022). In addition, pretreatment with a PCSK9 inhibitory peptide was found to be protective against brain damage in a rat model of cardiac ischemia/reperfusion injury (Apaijai et al., 2019). PCSK9 interacts with several proteins with known roles in neuronal survival, differentiation, migration and plasticity, including amyloid precursor protein (APP), amyloid precursor-like protein 2 (APLP2), sortilin (DeVay et al., 2013; Gustafsen et al., 2014; Butkinaree et al., 2015; Fu et al., 2017), heparan sulfate proteoglycans (HSGP; Gustafsen et al., 2017), very low density lipoprotein receptor (VLDLR) and apolipoprotein receptor type 2 (ApoER2; Poirier et al., 2008). Moreover, recent studies indicate that PCSK9 protein levels are inreased in Alzheimer’s disease (AD; Zimetti et al., 2016; Picard et al., 2019), and positively correlated with depression score (Macchi et al., 2020). Furthermore, PCSK9 genetic variants were found to be associated with greater mood instability and higher neuroticism in the UK Biobank (Hay et al., 2022).

Taken together, altered PCSK9 activity could potentially affect brain function in three different ways: (1) the potent effect of PCSK9 on plasma LDL cholesterol could affect exchange with the CNS and its overall lipid composition, (2) PCSK9 expressed in the brain could directly affect local cholesterol metabolism, and (3) local PCSK9 could affect neuronal function through interactions with receptor systems, independently of its role in cholesterol metabolism. To address this, we studied the consequences of complete loss of PCSK9 and the concomitant change in circulating cholesterol on biochemical and functional properties of the mouse brain. Furthermore, we investigated the impact of altered PCSK9 expression in the developing brain on the architecture of the cerebral cortex.

## 2. Materials and methods

### 2.1. Primary neurons

Cortical and hippocampal neurons were prepared from P0 and cerebellar granule neurons (CGN) from P4 C57BL/6 J BomTac mouse pups, following dissection in ice-cold phosphate buffered saline (PBS). Dissected cerebral cortex, hippocampus or cerebellum was dissociated 30 min in Leibovitz’s L-15 media (Gibco) with 20 U/mL activated papain (Worthington Biochemical Corporation) and washed in DMEM (Lonza) containing 0.01 mg/mL DNaseI (Sigma) and triturated in Neurobasal-A medium (Gibco) with added supplements (B-27 Supplement (Gibco), 2 mM GlutaMAX (Gibco), 100 μg/mL Primocin (Invivogen) and 20 μM floxuridine +20 μM uridine (Sigma) and 0.01 mg/mL DNaseI (Sigma). To isolate the cerebellar granule neurons (CGN) additional sedimentation and washing steps were performed. First, the test-tube was left untouched for 2 min and thereafter the upper cell layer of cerebellar cells was collected and washed three times in Neurobasal-A medium. Next, for WB protocols, the cells were seeded in supplemented Neurobasal-A medium onto poly-L-lysine (Sigma-Aldrich) and laminin (Invitrogen) pre-coated 6-well cell culture dishes at a density of 2 million cells per well and cultured for 3 days at 37°C and 5% CO_2_. The survival and quality (assessed by the abundance of intact neuronal processes) was visually assessed on a daily basis. For cortical neurons, half of the medium was changed on first day *in vitro* (DIV 1). For CGN, all medium was replaced at DIV 1. Purified PCSK9 protein (10 nM or 100 nM) was added on DIV 1 when changing the culture medium. Cells were lysed at DIV 3 in TNE buffer (10 nM Tris-base, 150 mM NaCl, 1 mM EDTA, 1% NP40; pH 8) supplemented with cOmplete™ protease inhibitor cocktail (Roche).

For the assessment of neurite length and branching, hippocampal neurons were seeded in supplemented Neurobasal-A medium onto poly-L-lysine (Sigma-Aldrich) and laminin (Invitrogen) pre-coated coverslips at a density of 5,000 neurons per coverslip with or without PCSK9 (10 nM). The coverslips with hippocampal neurons were used for analysis on DIV 3. To determine cell morphology, hippocampal neurons were stained for β-tubulin III (β-tubulin mouse monoclonal antibody MAB3408; Millipore), and confocal images were obtained. The length and the branching of the neurons was subsequently assessed by image analysis using Imaris software (Bitplane, Zürich, Switzerland).

### 2.2. Production of recombinant PCSK9

The sequence of human *PCSK9* used here is identical to sequence of GenBank acc. no. CAC38896.1. The coding region was ligated into the pCpGfree-vitroNmcs expression vector and transformed into the *E. coli* strain GT115 encoding the *pir* gene (Invivogen). CHO-K1 cells stably transfected with *PCSK9* were grown as a suspension in Hybridoma-SFM medium (GIBCO) and then expanded to Celline CL 350 Bioreactor flasks (Integra) or in triple layer flasks (Thermo Fischer Scientific). The medium from PCSK9-expressing CHO cells was 1:1 in 25 mM Tris pH 7.4 and applied to two serial-connected 5 mL HiTrap QFF columns (GEhealthcare). The columns were washed with 25 mM Tris pH 7.4 and protein subsequently eluted with 25 mM Tris pH 7.4 and 1 M NaCl. The eluted sample was dialyzed against 25 mM Tris pH 7.4 and 5% (v/v) glycerol at 4°C. The dialyzed sample was applied to a 5 mL Heparin HP column (GEhealthcare) and protein was eluted in 1 mL fractions by a linear gradient over 40 mL from 0 to 1 M NaCl in 25 mM Tris pH 7.4. Fractions containing PCSK9 were concentrated and further purified by SEC on a Superdex200 increase 10/300 or 16/600 in either PBS or 25 mM Tris pH 7.4 and 150 mM NaCl. Samples were analyzed by SDS-PAGE and PCSK9 was concentrated to 5–8 mg/mL based on absorbance at 280 nm and flash-frozen in liquid nitrogen before storage at-80°C.

### 2.3. Western blotting

Proteins were separated using SDS-PAGE (NuPAGE® 4–12% Bis-Tris; Thermo Fischer Scientific) in MOPS SDS Running Buffer (NuPAGE®, Thermo Fischer Scientific) and transferred to nitrocellulose membrane using iBlot™ Gel Transfer Stacks (Thermo Fischer Scientific). Membranes were blocked in 5% skimmed milk buffer in TBST (0.05 Tris-base, 0.5 M NaCl with 0.1% Tween-20) and incubated with primary antibodies diluted in blocking buffer overnight at 4°C. On the next day, the membranes were washed three times with 0.5% skimmed milk in TBST, incubated with HRP-conjugated secondary antibodies for 1 h at room temperature (RT), and washed again 5 times. Proteins were visualized using ECL plus Western blotting detection system (GE Healthcare) with a Fuji film LAS3000 system. The following antibodies were used: Rabbit anti-LDLR (Abcam ab52818, 1:600), Mouse anti-beta actin (Sigma A5441, 1:5000). Secondary HRP-linked antibodies were used in 1:2000 dilution (Dako P0260, Cell signaling 7074S).

### 2.4. PCSK9 knockout mice

The PCSK9 knockout (KO) mice (B6; 129S6-*Pcsk9*tm1Jdh/J; Strain #005993) were purchased from Jackson Laboratory and backcrossed for 10 generations to C57BL/6 J Bomtac background. The KO mice were subsequently compared to the same C57BL/6 J Bomtac substrain used for backcrossing. The backcrossed PCSK9 homozygous KO mice were used in all further experiments.

### 2.5. ELISA

Tissue PCSK9 and LDLR concentrations were measured using Quantikine ELISA kits (MPC900, MLDLR0) from R&D Systems according to the manufacturer’s protocol. In brief, for analysis of LDLR levels, adult mouse cerebellum (3 to 12 months old) was removed in ice cold PBS and snap-frozen. Liver tissue and cerebellum tissue for assessment of PCSK9 levels were removed from PBS perfused adult WT and PCSK9 KO animals (14–15 week-old) and snap-frozen. The isolated tissues were subsequently homogenized in TNE buffer supplemented with cOmplete™ protease inhibitor cocktail (Roche).

### 2.6. *In utero* electroporation

For IUE, the PCSK9 coding sequence was derived from the Origene clone RC2200000, the C-terminal FLAG-tag was eliminated and a NotI/EcoRI was fragment was ligated into the pCAGEN vector (Addgene #11160) and sequenced. The PCSK9 coding sequence is identical to the sequence in GenBank acc no.: CAC38896.1. We validated the PCSK9 plasmid using HEK293 cell line. We transfected the cells with the PCSK9 plasmid or an empty pCAGEN vector and stained with an anti-PCSK9 antibody (Goat-a-PCSK9: AF3985 (anti mouse/rat PCSK9, 0.2 mg/mL), dilution 1:50) and used immunofluorescence to confirm specific PCSK9 expression. pCAG-GFP was purchased from Addgene #11150. pCX::myr-Venus plasmid was a gift from Anna-Katerina Hadjantonalis.

Time mated pregnant WT Naval Medical Research Institute (NMRI) mice (2–6 month old) were used (Charles Rivers and Janvier Labs RRID:IMSR TAC:nmri). The mice were housed at the animal facility of Charité University Hospital in compliance with the guidelines of Landesamt für Gesundheit und Soziales (LaGeSo), permission number G0054/19. Experiments were performed as described in Saito (2006). The DNA mixture (500 ng/μL of total DNA and 0.1% of Fast Green Dye (Sigma-Aldrich)) was injected into lateral ventricle of the mouse pups at E12.5 and electroporated into the ventricular cells using platinum electrodes, 6 pulses of 37 V were applied, pulse length was 50 ms and pulse interval 950 ms. Thereafter, the pups were returned into the abdominal cavity of the pregnant dam. After 96 h, the dam and the pups were sacrificed, and the brains of treated mice were isolated and fixed.

### 2.7. Immunohistochemistry

Embryonic brains were fixed overnight at 4°C in 4% paraformaldehyde in PBS, washed in PBS and incubated sequentially in 10 and 30% sucrose solutions in PBS until sinking to the bottom of the container. Thereafter, the brains were frozen in −38 to −40°C isopentane. 50 μM coronal brain cryosections were prepared using Leica CM3050S cryostat and collected in PBS/0.01% sodium azide solution.

Floating brain sections were washed three times with PBS at room temperature and incubated with blocking solution (5% horse serum, 0.5% (v/v) Triton X-100, PBS) for 30 min at RT with gentle agitation. Next, the brain sections were incubated with the primary antibody and DRAQ5™ (Biostatus Limited, 1:2000) diluted in blocking buffer for 16 to 20 h at 4°C with gentle agitation, washed in PBS three times for 15 min and incubated with secondary antibody coupled to a fluorophore of choice for 4 h at RT, with gentle agitation. Thereafter, sections were transferred to PBS and collected to a Superfrost Plus (Thermo Fisher Scientific) glass slide, dried and mounted with cover glass (Menzel-Gläser) and Immu-Mount mounting medium (Shandon, Thermo Fisher Scientific). The following antibodies were used: Goat anti-GFP (600–101-215 M, Rockland, 1,1,000), Rabbit anti-Satb2 (generated in-house, 1:300), Rat anti-Ctip2 (25B6, Abcam, 1,300). Fluorophore 488-, Cy3-, and Cy5-conjugated secondary antibodies (Jackson ImmunoResearch, 1:300).

### 2.8. Image acquisition and analysis

For imaging, Leica SL confocal microscope with 40X objective was used and 2.5 μM thick z-stacks were taken of each coronal brain slice. Stitching of the images after acquisition was performed using Fiji (ImageJ) Pairwise Stitching Plugin. The maximum height of the cortical stacks was divided into 5 bins of identical dimensions and the number of immunostained/transfected neurons in each bin was counted manually using the Cell Counter plug-in in Fiji (ImageJ) software. Localization of cell fate markers was also analyzed using the Cell Counter Plugin in Fiji (ImageJ) software.

### 2.9. Lipid extraction and analysis

Male mice (13-to 15-weeks of age) were used for lipidomics analysis. The mice were euthanized by cervical dislocation; cerebellum and cerebral cortex were dissected in ice-cold LC–MS grade water and snap-frozen. All dissection tools were washed in absolute ethanol and new tools and glass petri dishes were used for each brain sample to avoid cross-contamination of lipids.

Chemicals, solvents, and synthetic lipid standards were purchased from Sigma-Aldrich (St. Louis, MO, United States), Rathburn Chemicals (Walkerburn, Scotland), Avanti Polar Lipids (Alabaster, AL, United States), and VWR (Soborg, Denmark). Cerebral cortex and cerebellum were homogenized in 155 mM ammonium bicarbonate and analyzed for total protein concentration using BCA Protein Assay Kit (Thermo Fisher Scientific). Aliquots corresponding to 30 μg of total protein brain tissue homogenate in 200 μL 155 mM ammonium bicarbonate were subjected to lipid extraction by a modified Bligh and Dyer protocol executed at 4°C (Zech et al., 2009; Nielsen et al., 2020). Briefly, the samples were spiked with 15 μL internal lipid standard mixture containing: 45 pmol cholesteryl ester (CE) 15:0-D_7_, 30 pmol ceramide (Cer) 18:1;2/12:0;0, 300 pmol cholesterol (FC)-D_4_, 15 pmol diacylglycerol (DAG) 12:0/12:0, 18.5 pmol dihexosylceramide (diHexCer) 18:1;2/17:0;0, 22.5 pmol monosialotetrahexosylganglioside (GM1) 36:1;2-D3, monosialotrihexosylganglioside (GM2) 36:1;2-D3, monosialodihexosylganglioside (GM3) 36:1;2-D3, 37.5 pmol hexosylceramide (HexCer) 18:1;2/12:0;0, 37.5 pmol lysophosphatidic acid (LPA) 17:0, 30 pmol lysophosphatidylcholine (LPC) 12:0, 37.5 pmol lysophosphatidylethanolamine (LPE) 13:0, 22.5 pmol lysophosphatidylglycerol (LPG) 17:1, 30 pmol lysophosphatidylinositol (LPI) 13:0, 30 pmol lysophosphatidylserine (LPS) 17:1, 37.5 pmol phosphatidic acid (PA) 12:0/12:0, 37.5 pmol phosphatidylcholine (PC) 12:0/12:0, 37.5 pmol phosphatidylethanolamine (PE) 12:0/12:0, 22.5 pmol phosphatidylglycerol (PG) 12:0/12:0, 22.5 pmol phosphatidylinositol (PI) 8:0/8:0, 30 pmol phosphatidylserine (PS) 12:0/12:0, 30 pmol sphingomyelin (SM) 18:1;2/12:0;0, 30 pmol sulfatide (SHexCer) 18:1;2/12:0;0, 15 pmol triacylglycerol (TAG) 17:0/17:0/17:0, 30 pmol trihexosylceramide (triHexCer) 18:1;2/17:0;0. Next, 1,000 μL of chloroform/methanol (2,1, v/v) was added to the samples and shaken at 2,000 rpm for 20 min. Thereafter, the samples were centrifuged for 2 min at 500 g. The lower organic phase was transferred to a new tube and vacuum-evaporated for 60 min. The lipids were dissolved in 75 μL chloroform/methanol (1,2, v/v).

Lipid analyses were performed with quantitative mass spectrometry-based shotgun lipidomics as described previously (Nielsen et al., 2020) with minor modifications. In brief, lipid extracts were subjected to MS analysis using a Q Exactive Hybrid Quadrupole-Orbitrap (quadrupole-Orbitrap) mass spectrometer (Thermo Fisher Scientific) equipped with a TriVersa NanoMate (Advion Biosciences, Ithaca, NY, United States). Lipid extracts of 10 μL were mixed with 12.9 μL 13.3 mM ammonium acetate in 2-propanol or with 10 μL 0.2% methylamine (v/v) in chloroform/methanol (1,5, v/v) for positive and negative ionization mode analysis, respectively. For the positive ionization mode analysis, samples were infused using a backpressure of 1.25 psi and ionization voltage of 0.95 kV. For the negative ionization mode analysis, samples were infused using a backpressure of 0.7 psi and ionization voltage of −1.06 kV. All data were recorded using FT MS and FT MS/MS scans in the positive and negative ionization modes as described previously (Nielsen et al., 2020).

Annotation of lipids are as previously described (Shevchenko and Simons, 2010; Liebisch et al., 2013; Nielsen et al., 2020). The glycerolipid (GL) and glycerophospholipid (GPL) species are annotated according to their sum composition: <lipid class> < total number of C in fatty acid moieties>: <total number of double bonds in fatty acid moieties> (e.g., PE 34:1). The SL species are also annotated according to their sum composition: <lipid class> < total number of C in the long-chain base and fatty acid moiety>: <total number of double bonds in the long-chain base and fatty acid moiety>; <total number of OH groups in the long-chain base and fatty acid moiety> (e.g., Cer 34:1;2).

Nomenclature abbreviations: Cer, ceramide; FC, cholesterol; DAG, diacylglycerol; GM1, monosialotetrahexosylganglioside; GM3, monosialodihexosylganglioside; HexCer, hexosyl ceramide (GlcCer or GalCer); LPA, lysophosphatic acid; LPC, lysophosphatidylcholine; LPE, lysophosphatidylethanolamine; LPG, lysophosphatidylglycerol; LPI lysophosphatidylinositol; LPS, lysophosphatidylserine; PA, phosphatidic acid; PC, phosphatidyl choline; PE, phosphatidyl ethanolamine; PG, phosphatidylglycerol; PI, phosphatidylinositol; PS, phosphatidylserine; SM, sphingomyelin; SHexCer, sulfatide. The variations of LPA, LPC, LPE, LPG, LPI, LPS, PA, PC, PE, PG, PI, and PS with O-in the end are ether lipids, where the hydrocarbon chain at the *sn-1* position of the glycerol backbone is attached by an ether bond as opposed to an ester bond (Dean and Lodhi, 2018).

LipidXplorer was used to report the *m/z* values and intensities of lipid species and their fragment ions of the acquired FT MS and FT MS/MS data (Herzog et al., 2012, 2013). The absolute molar lipid quantities were calculated using an in-house built R-based suite of scripts named LipidQ^1^ based on the reported intensities of sample-derived lipids and of internal lipid standards. The relative molar quantities based on the absolute quantities of individual lipids and their sum was calculated with LipidQ (Han and Gross, 2005; Ejsing et al., 2006, 2009; Sampaio et al., 2011). Principal components analyses were performed using ClustVis web tool.^2^ The source code of ClustVis is available in GitHub.^3^

### 2.10. Behavioral studies

All behavioral experiments were approved by the Danish Animal Experiments Inspectorate (2016-15-0201-01127) and carried out according to institutional and national guidelines. All mice were bred and housed at the Animal Facility at the Department of Biomedicine, Aarhus University.

*Barnes maze* - Spatial memory and learning was assessed using the Barnes maze paradigm. The maze (PanLab Harvard Apparatus) consisted of a circular platform (92 cm in diameter and 105 cm high) and had 20 equally spaced holes (5 cm in diameter and 7.5 cm a part). The target hole had a hidden escape box attached to it. Visual cues on the wall helped the mice with spatial orientation and a ventilator with loud noise motivated the mice to escape from the open area faster. On the first day, the mice were placed in the center of the maze under a dark circular bowl to ensure random orientation. They were allowed to freely explore the maze for 3 min. In case they did not find the escape hole, the experimenter guided them there manually. Mice were tested for five consecutive days and their latency to enter the target hole and time to target were measured by video recording and analysis using the Any-Maze tracking Software (Stoelting, Dublin, Ireland).

*Rotarod* - Motor coordination and motor skill learning were evaluated in the rotarod test, using the rotating rod apparatus (LE8200, PanLab Harvard Apparatus). Mice were placed on the rod (3-cm-diameter) for three trials per day over four consecutive days, and their latency to fall from the rod was recorded. The rotating rod accelerated from 4 to 40 rpm across a 5-min period with linear speed development. Best trial of round 1 on each day was evaluated. If the mice fell down because of misplacement or if they were turning around or grooming, they were placed on the rotarod again and the time was reset. The mice were placed on the rod a maximum of 10 times per session on day 1 while for the subsequent days as follows; 10 times for the first session and 3 times during the following training rounds. Mice rested a minimum of 20 min between sessions to avoid fatigue and exhaustion.

*Open field* - For assessment of general locomotion, exploration of a novel environment and anxiety-related behavior, the animals were tested in the open field test (PanLab Harvard Apparatus) consisting of a (40x40x35 cm) clear Plexiglas arena. Mice were placed in the corner of the arena and their activity was recorded for 20 min. The total distance travelled, entries to the center zone, time spent in the center zone and distance traveled in the center zone was analyzed using the Any-Maze tracking Software (Stoelting, Dublin, Ireland).

*Elevated plus maze* - Elevated plus maze was used to assess exploration of a novel environment and anxiety-related behavior. The maze (PanLab Harvard Apparatus) is 40 cm above the floor and consists of two opposite enclosed arms with 15 cm high opaque walls and two opposite open arms of the same size (35×5 cm). The test was carried out in a dimly lit room under a video camera and analyzed using the Any-Maze tracking system. Ten-minute testing sessions were carried out for each mouse, and the number of entries and time spent in open arms were measured. The percentage of entries into the open arms was calculated as open arm entries/(open arm entries + closed arm entries) x100.

*Marble burying* - compulsive behavior was assessed using the marble burying test. Twelve colored marbles (15 mm diameter) were placed in three rows on top of the wood chip bedding. A marble was considered buried when covered by more than two-thirds of their diameter. Animals were tested for 20 min and the number of buried marbles was counted at the end of the test by two observers blinded to the genotype.

### 2.11. Statistics

Graph Pad Prism 8 (GraphPad Software) was used for the analysis of the data. Statistical significance was evaluated using a two-tailed Student’s *t*-test or two-way ANOVA as indicated in the figure legends and supplementary tables. Data are presented as mean ± standard errors of the mean (SEM) as indicated. *p* values below 0.05 were considered statistically significant.

## 3. Results

### 3.1. PCSK9 Induces low-density lipoprotein receptor degradation in cultured primary neurons

**Figure 1:**
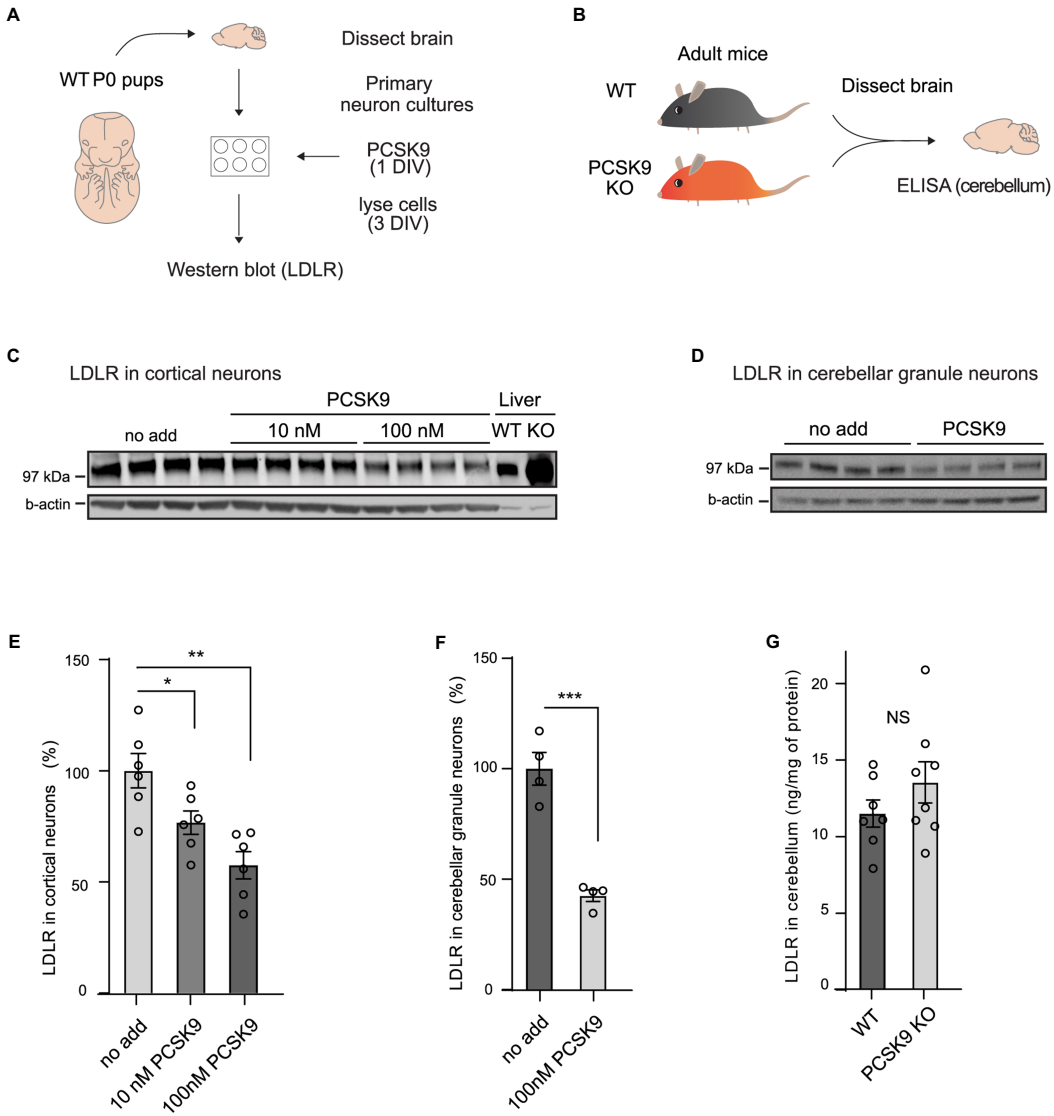
PCSK9 targets LDLR in neuronal cell cultures but not in adult cerebellum. **(A)** Schematic representation of the experiment with PCSK9 treatment on primary neuronal cultures. **(B)** Schematic representation of the assessment of the LDLR levels in the PCSK9 KO mouse cerebellum using ELISA. Representative Western blots of LDLR receptor in cortical **(C)** and cerebellar granule neurons **(D)**. Primary cultures were treated with 10 nM or 100 nM concentration of PCSK9 at 1  day *in vitro* (DIV) and lysed at 3 DIV. Liver samples of WT and PCSK9 KO mice were added as control **(C)**. Densitometric quantification of LDLR normalized to β-actin in cortical (*n* = 6; **E**), and cerebellar granule neurons (*n* = 4; **F**). ELISA of LDLR in cerebellum (*n* = 7 WT, 8 KO) of adult mice (3- to 12-month-old; **G**). Data are represented as mean ± SEM. Statistical significance was evaluated using a two-tailed Student’s t-test, alpha = 0.05. Asterisks indicate significantly different values (NS *p* > 0.05; *p ≤ 0.05; **p ≤ 0.01; ***p ≤ 0.001).

To study if PCSK9 directly affects receptor levels and cellular processes in neurons, we first analyzed LDLR levels in cultured primary neurons (Figures 1A,C–F; Supplementary Figure S1A,B). We found that LDLR levels dropped significantly in response to exogenous PCSK9 (10 nM and100nM) in cortical (Figures 1C,E), cerebellar granule neurons (CGN; Figures 1D,F), and in hippocampal neurons (Supplementary Figures S1A,B), suggesting that the molecular machinery necessary for PCSK9-induced LDLR degradation is present in neuronal cells. In addition, we found that exogenous PCSK9 (10 nM) affected the neurite length and the branching of hippocampal neurons (Supplementary Figures S1C–E). This demonstrates that PCSK9 can regulate neuronal processes such as growth and branching *in vitro*. However, no significant change in LDLR was observed in homogenates of adult PCSK9 KO cerebellum (13.5 ng/mg LDLR protein ±3.79 SEM) compared to WT control tissue (11.5 ng/mg LDLR protein ±2.33 SEM; Figures 1B,G) using ELISA. Considering that lack of PCSK9 resulted in a marked increase in LDLR in the liver (Figure 1C), this suggests that LDLR is differentially regulated in the brain and periphery.

### 3.2. PCSK9 does not influence neuronal migration and cortical development

PCSK9 is highly expressed in mouse and human developing cerebral cortex (Seidah et al., 2003; brainspan.org). In mouse brain, the expression is highest around E12.5 (Seidah et al., 2003) – a time point where cortical development and migration of cortical neurons are peaking (Mukhtar and Taylor, 2018). Interestingly, PCSK9 is known to induce degradation of VLDLR and ApoER2 in *in vitro* systems (Poirier et al., 2008). These proteins are key signaling receptors in the Reelin pathway (Poirier et al., 2008; Roubtsova et al., 2011; Sekine et al., 2014) and hence critical for proper cortical and cerebellar development as well as neuronal plasticity (Trommsdorff et al., 1999; Armstrong et al., 2019). We therefore studied the role of PCSK9 in neuronal cell fate and cortical layering of the embryonic brain. Cortical layering is a tightly controlled process that occurs in an inside-out manner, meaning that newborn neurons migrate through the layers of earlier born neurons and thus the earlier born neurons form the deeper layers and later-born neurons form the upper layers (Mukhtar and Taylor, 2018). The specific neuronal subtypes residing in distinct layers can be classified according to their location and expression of specific markers (Mukhtar and Taylor, 2018).

**Figure 2:**
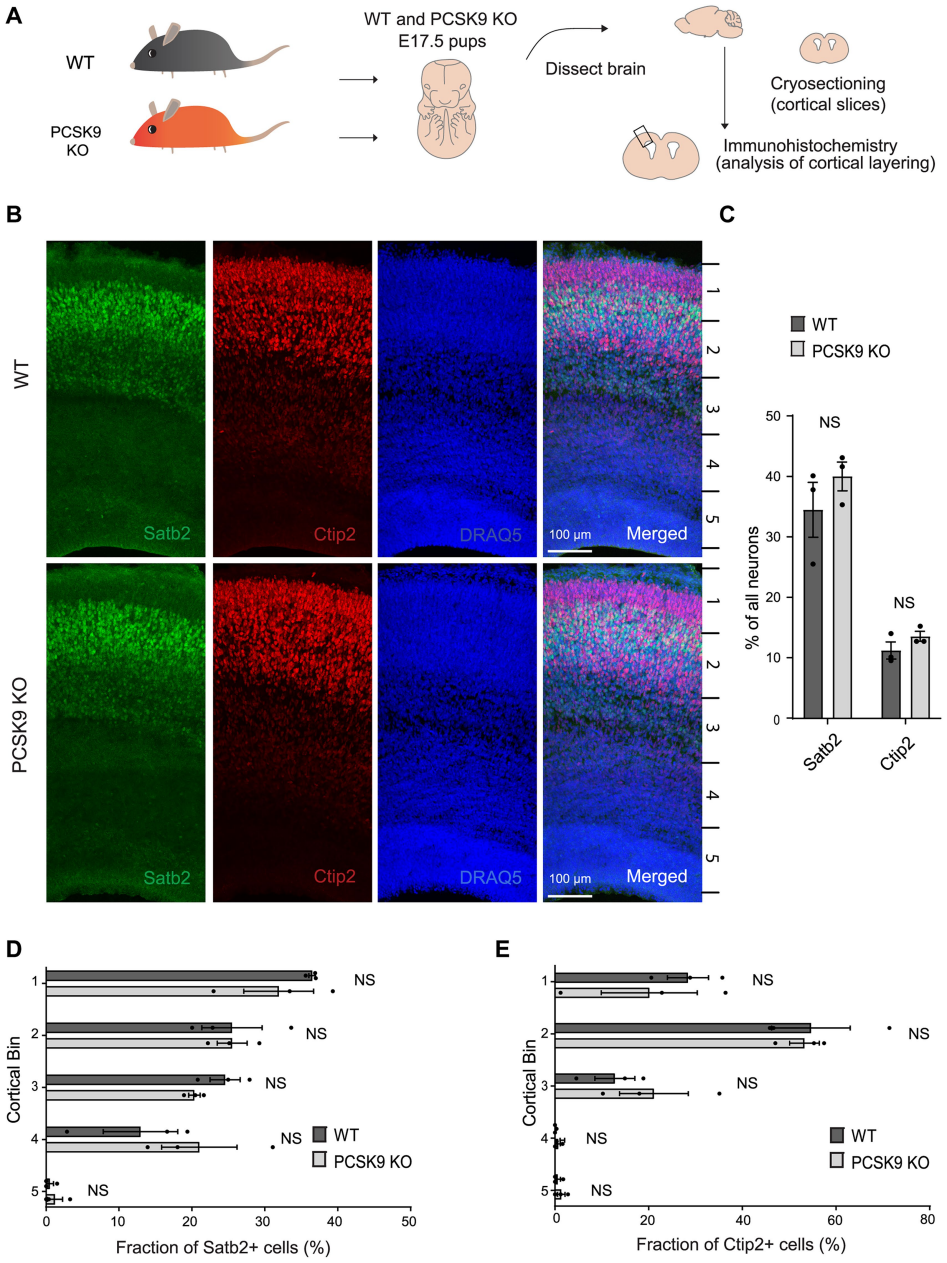
Lack of PCSK9 during development does not affect the fate of cortical progenitor cells. **(A)** Schematic representation of the experiment. **(B)** Representative images of immunostaining signals (Satb2, Ctip2, and DRAQ5) in WT and PCSK9 KO E17.5 cortices. **(C)** Quantification of the fraction of all neurons (DRAQ5 labelled) expressing indicated fate markers (Satb2, Ctip2) in WT and PCSK9 KO E17.5 cortical slices (see also Supplementary Table S1A). **(D,E)** Quantification of laminar distribution of cortical neurons labelled with Satb2 **(D)** or Ctip2 **(E)** fate markers in WT and PCSK9 KO E17.5 cortical slices (see also Supplementary Table S1B). Cortical bin – the maximum height of the cortical slice was divided into 5 bins of identical dimensions. Results on graphs are represented as average percent ± SEM. For statistical analyses two-way ANOVA with Bonferroni multiple comparison test was performed, alpha = 0.05. All *p* values were above 0.05 and considered non-significant; *n* = 3 WT, 3 KO.

First, we immunostained cortical slices from PCSK9 KO and WT mice at embryonic day 17.5 (E17.5) with layer specific markers: special AT-rich sequence-binding protein 2 (Satb2) that labels neocortical post-mitotic upper-layer neurons, and chicken ovalbumin upstream promoter transcription factor-interacting protein 2 (Ctip2) that is expressed by deeper layer neurons in the neocortex (Figure 2; Supplementary Tables S1A,B; Britanova et al., 2006; Mukhtar and Taylor, 2018). *Pcsk9* expression in telencephalon is reported to peak around E12.5 (Seidah et al., 2003) and disturbances in cell fate or migration during this period are expected to result in apparent changes at later stages (e.g., E17.5). However, we did not observe any aberrancies in the cell fate judging from the amount of upper and deeper layer neurons that express Satb2 or Ctip2 markers, respectively (Figures 2B,C; Supplementary Table S1A). In addition, the distribution of the labeled neurons in the E17.5 PCSK9 KO cerebral cortex resembled those of WT mouse cerebral cortex (Figures 2B,D,E and Supplementary Table S1B). Thus, we conclude that lack of PCSK9 during development does not affect cell fate or cortical layering.

We next studied the consequences of PCSK9 overexpression on cortical layering by transfection of cortical progenitors of WT mice at E12.5 with a PCSK9 encoding plasmid (pCAG-PCSK9) together with pCAG-GFP and pCX::myr-Venus co-expression vectors using *in utero* electroporation (IUE), and subsequently analyzed brain slices at E16.5 (Supplementary Figure S2). As a negative control, we expressed plasmids encoding GFP, Venus, and an empty pCAG vector in neuronal progenitors of WT mouse embryos at E12.5 (Supplementary Figure S2; Supplementary Tables S1C,D). GFP-positive neurons in both PCSK9 transfected and negative controls distributed similarly and as expected from their birth date at E12.5 (Supplementary Figures S2B,C; Supplementary Table S1C). Moreover, the fate of both upper-and deeper-layer neurons was unchanged when compared to the control (Supplementary Figures S2B,D; Supplementary Table S1D). In summary, these data show that neither the absence nor excess of PCSK9 in the developing brain at the critical period of laminar development interferes with the migration or fate of progenitor cells *in vivo.*

### 3.3. PCSK9 deficiency results in compensatory lipid changes in adult mouse brain

**Figure 3:**
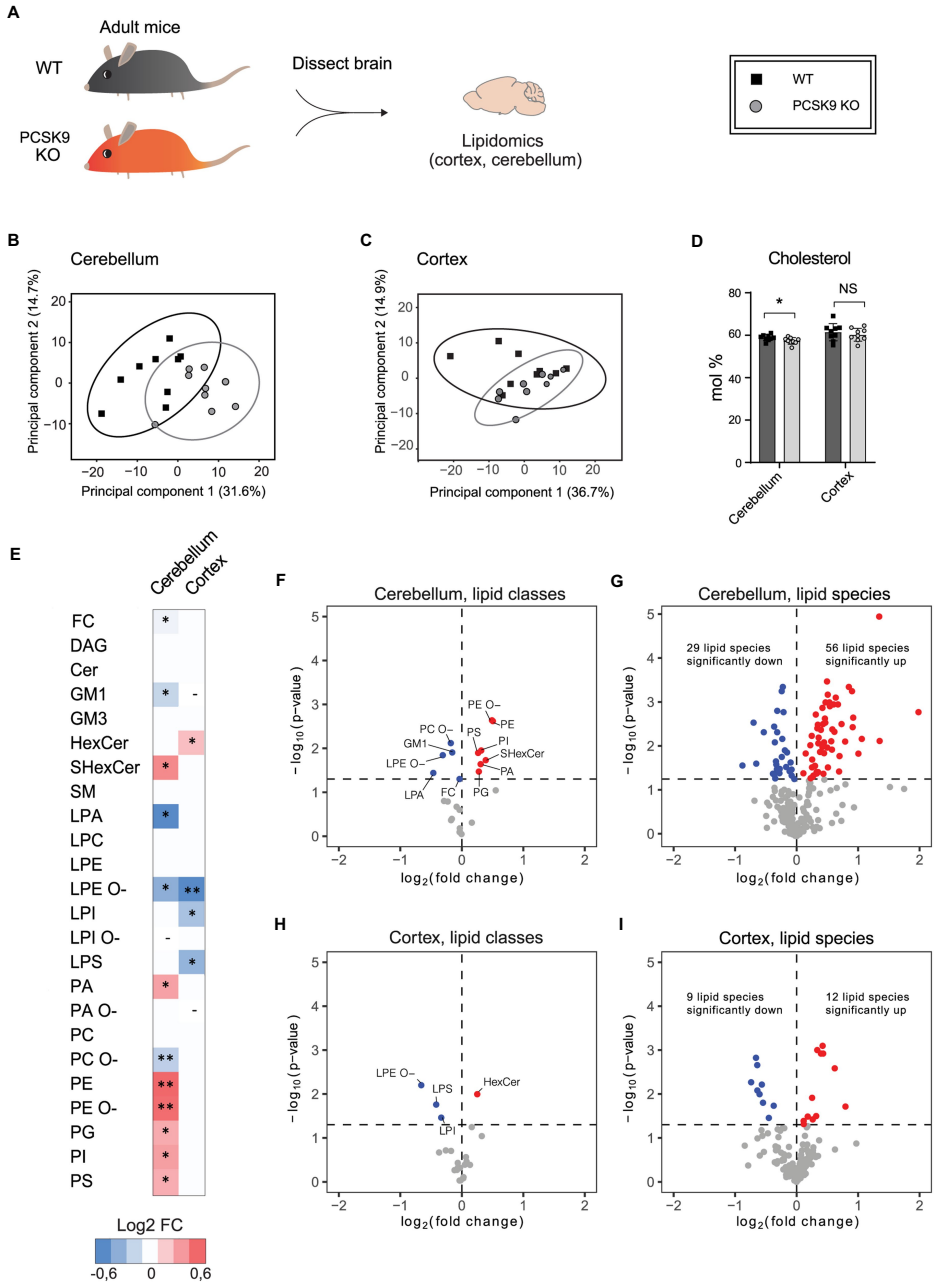
The effect of PCSK9 deficiency on lipids in cerebellum and cortex. Lipid profiles of mouse cerebellum and cortex were analyzed using a quantitative mass-spectrometry based shotgun lipidomics method. **(A)** Schematic representation of the experiment. **(B,C)** Principal component analysis showing different clustering of WT and KO cerebellar **(B)** and cortical **(C)** lipidomes. **(D)** Cholesterol (FC) content in mol% in WT and (Continued)FIGURE 3 (Continued)PCSK9 KO cerebellum and cortex. Data are represented as mean ± SEM. **(E)** Heatmap of different lipid classes in PCSK9 KO cortex and cerebellum in mol % compared to WT, shown as log2 fold changes. **(F–I)** Volcano plots representing the significantly up- and down-regulated lipid classes and species in cerebellum **(F,G)** and cortex **(H, I)**. *N* = 9 per group; 13-to 15-week-old male mice. Only statistically significant results showed. Statistical significance was assessed using unpaired two-tailed Student’s t test, alpha = 0.05. See also Tables 1, 2 for descriptive statistics and individual p values. Asterisks indicate significantly different values (*p ≤ 0.05; **p ≤ 0.01). Dash indicates the absence the lipid class in the indicated brain area. The variations of LPA, LPC, LPE, LPG, LPI, LPS, PA, PC, PE, PG, PI, and PS with O-in the end are ether lipids.

**Figure 4:**
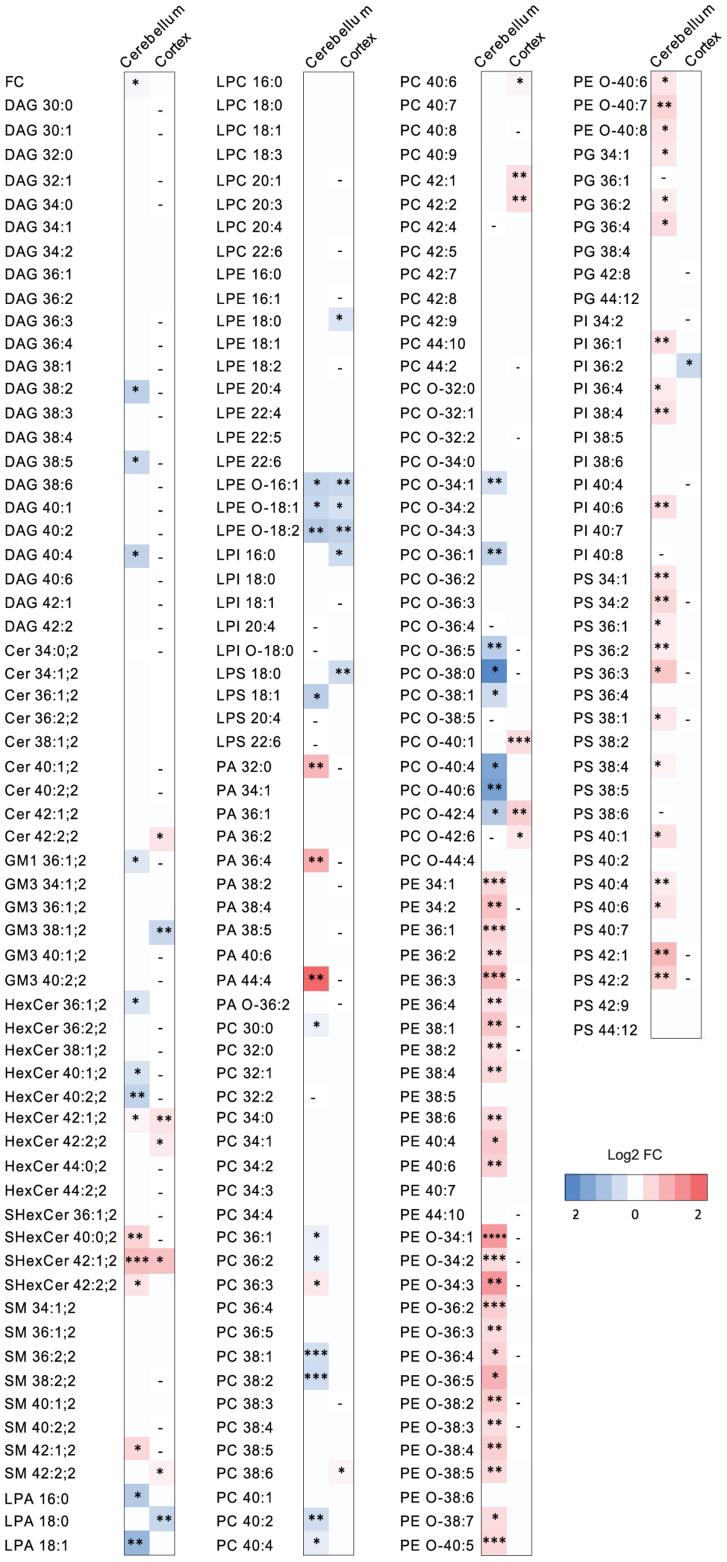
The effect of PCSK9 deficiency on specific lipid species in cerebellum and cortex. Lipid profiles of mouse cerebellum and cortex were studied using a quantitative mass-spectrometry based shotgun lipidomics method. Heatmap of lipid species in PCSK9 KO cortex and cerebellum in mol % compared to WT, shown as log2 fold changes. *N* = 9 per group; 13-to 15-week-old male mice. Only statistically significant results showed. Statistical significance was assessed using unpaired two-tailed Student’s *t* test, alpha = 0.05. See also Supplementary Tables S2, S3 for descriptive statistics and individual p values. Asterisks indicate significantly different values (*p ≤ 0.05; **p ≤ 0.01; ***p ≤ 0.001; ****p ≤ 0.0001). Dash indicates the absence of the lipid species in the brain area.

PCSK9 plays a major role in peripheral lipid homeostasis and is known to affect plasma lipid and lipoprotein particle composition beyond cholesterol (Soutar, 2011; Jänis et al., 2013; Hilvo et al., 2018). Hence, to understand how PCSK9 influences brain lipid composition, we performed lipidomic profiling of WT and PCSK9 KO cerebellum and cerebral cortex at 13–15 weeks of age. Using quantitative mass spectrometry-based shotgun lipidomics (Nielsen et al., 2020), we detected 23 lipid classes covering 218 species in cerebellum (Figures 3, 4; Table 1; Supplementary Table S2) and 22 lipid classes covering 160 species in cerebral cortex (Figures 3, 4; Table 2; Supplementary Table S3). Lipid classes are defined by a common head group, whereas varying acyl chain length, unsaturation and hydroxylation determine distinct lipid species within a class (Harayama and Riezman, 2018).

**Table 1:** Lipid classes detected in WT and KO cerebellum.

	Average mol% ± SEM		
	WT	PCSK9 KO	*p* value
FC	58.806 ± 0.472	57.325 ± 0.515	0.0499
DAG	0.774 ± 0.061	0.755 ± 0.049	0.8077
Cer	0.254 ± 0.033	0.284 ± 0.027	0.4911
GM1	0.218 ± 0.006	0.195 ± 0.006	0.0124
GM3	0.075 ± 0.003	0.074 ± 0.002	0.6604
HexCer	3.426 ± 0.132	3.231 ± 0.073	0.2137
SHexCer	0.410 ± 0.035	0.537 ± 0.034	0.0186
SM	2.152 ± 0.033	2.145 ± 0.039	0.8949
LPA	0.034 ± 0.004	0.025 ± 0.002	0.0363
LPC	0.362 ± 0.029	0.309 ± 0.022	0.1625
LPE	1.150 ± 0.126	1.028 ± 0.066	0.4035
LPE O-	0.036 ± 0.002	0.029 ± 0.001	0.0143
LPI	0.038 ± 0.004	0.034 ± 0.003	0.4323
LPS	0.134 ± 0.014	0.110 ± 0.009	0.1579
PA	0.226 ± 0.011	0.279 ± 0.018	0.0229
PA O-	0.002 ± 0.000	0.004 ± 0.001	0.0904
PC	24.557 ± 0.405	23.939 ± 0.324	0.2516
PC O-	0.362 ± 0.012	0.320 ± 0.007	0.0076
PE	2.342 ± 0.171	3.323 ± 0.212	0.0024
PE O-	2.192 ± 0.144	3.080 ± 0.198	0.0023
PG	0.077 ± 0.004	0.093 ± 0.006	0.0338
PI	0.704 ± 0.036	0.877 ± 0.048	0.0112
PS	1.670 ± 0.068	2.008 ± 0.099	0.0127

**Table 2:** Lipid classes detected in WT and KO cortex.

	Average mol% ± SEM		
	WT	PCSK9 KO	*p* value
FC	61.421 ± 1.354	60.202 ± 1.009	0.4810
DAG	0.254 ± 0.029	0.251 ± 0.013	0.9222
Cer	0.775 ± 0.020	0.866 ± 0.040	0.0567
GM3	0.048 ± 0.001	0.046 ± 0.002	0.3702
HexCer	0.901 ± 0.033	1.072 ± 0.049	0.0101
SHexCer	0.087 ± 0.008	0.108 ± 0.010	0.0908
SM	0.888 ± 0.019	0.931 ± 0.032	0.2729
LPA	0.029 ± 0.002	0.024 ± 0.003	0.1920
LPC	0.194 ± 0.010	0.197 ± 0.009	0.7796
LPE	0.505 ± 0.016	0.483 ± 0.024	0.4705
LPE O-	0.020 ± 0.002	0.013 ± 0.002	0.0063
LPI	0.043 ± 0.003	0.034 ± 0.002	0.0345
LPI O-	0.004 ± 0.001	0.004 ± 0.001	0.9319
LPS	0.159 ± 0.011	0.119 ± 0.010	0.0174
PA	0.070 ± 0.010	0.054 ± 0.007	0.2142
PC	26.663 ± 0.802	27.599 ± 0.750	0.4067
PC O-	0.394 ± 0.018	0.416 ± 0.013	0.3440
PE	2.680 ± 0.190	2.727 ± 0.091	0.8274
PE O-	1.797 ± 0.192	1.971 ± 0.070	0.4101
PG	0.062 ± 0.003	0.055 ± 0.004	0.1974
PI	0.785 ± 0.087	0.723 ± 0.048	0.5415
PS	2.224 ± 0.140	2.106 ± 0.120	0.5343

The WT and PCSK9 KO lipid profiles of the cerebellum clustered separately, as illustrated by principal components analysis of all samples (Figure 3B). A small but significant decrease in the relative cholesterol (FC) content from 58.8 mol% in WT mice to 57.3 mol% was observed in the KO mice (Figures 3D–F; Table 1). Pronounced changes were found in glycerophospholipids (GPL), including significant changes in phosphatidyl ethanolamine (PE) and PE ether (PE O-), which were increased by more than 40% at class level and up to 87% (PE 36:3) and 155% (PE O-34:3) at species levels; and phosphatidylserine (PS), phosphatidylglycerol (PG), phosphatidylinositol (PI), phosphatidic acid (PA), which increased by more than 20% at class level and up to 295% at species levels (PA 44:4; Figures 3E–G; Figure 4; Table 1; Supplementary Table S2). In fact, almost all of the detected species of PE and PE O-, most of the PS species, and several of the PG, PI and PA species were significantly increased (Figure 4; Supplementary Table S2). At the same time, several GPL classes decreased significantly: phosphatidyl choline ether (PC O-) decreased by 12%, lysophosphatidylethanolamine (LPE) ether (LPE O-) by 19% and lysophosphatic acid (LPA) decreased by 27% in the KO cerebellum compared to WT (Figures 3E–G, 4; Table 1; Supplementary Table S2). There were no overall changes in phosphatidyl choline (PC) levels, which is the most abundant lipid in the CNS next to cholesterol (Figures 3E–F; Table 1). At the same time, several PC species with a low number of double bonds in acyl chains (30:0, 36:1, 36:2, 38:1, 38:2 and 40:2) and a polyunsaturated species (40:4), dropped significantly between 5 and 16% (Figure 4; Supplementary Table S2). PC 36:3 levels, on the other hand, increased by 20% (Figure 4; Supplementary Table S2). Diacylglycerol (DAG) class levels remained unchanged (Figures 3E,F; Table 1), while three out of 23 detected DAG species (38,2, 38:5, 40:4) were significantly reduced in the KO by at least 17% (Figure 4; Supplementary Table S2). Generally, these findings indicate that PCSK9 deficiency affects the length of acyl chains and the degree of unsaturation of several PC and DAG species in cerebellum. Notably, those properties determine the specific role and position of the lipid in the lipid membrane.

Lack of PCSK9 also lead to changes in sphingolipid (SL) classes and species in the cerebellum. We observed a 10% reduction in the levels of monosialotetrahexosylganglioside (GM1), which is one of the most abundant gangliosides in the brain, and a 31% increase in the levels of sulfatide (SHexCer) including most of the detected species (Figures 3E–G, 4 Table 1; Supplementary Table S2). Three out of four detected SHexCer species (40:0;2, 42:1;2, 42:2;2) were significantly increased up to 80% (Figure 4; Supplementary Table S2). Even though there were no statistically significant changes in hexosyl ceramide (GlcCer or GalCer) (HexCer) and sphingomyelin (SM) classes (Figures 3E,F; Table 1), certain HexCer species (36:1;2, 40:1;2, 40:2;2) were markedly reduced by up to 19%, whereas HexCer 32:1;2 and SM 42:1;2 were increased up to 42%, respectively (Figure 4; Supplementary Table S2), in the KO cerebellum. Thus, PCSK9 deficiency seems to affect SL acyl chain pattern and the degree of unsaturation.

Lipid changes were less obvious in the cerebral cortex of PCSK9 KO animals (Figures 3C–E,H,I, 4), although the levels of several lipid classes and species were significantly altered (Figures 3E,H,I, 4; Table 2; Supplementary Table S3). Various GPL classes such as lysophosphatidylinositol (LPI) (including LPI 16:0), lysophosphatidylserine (LPS) and LPE O- (including LPE O-species 16:1, 18:1, 18:2) were reduced up to 37% compared to WT (Figures 3E,H, 4; Table 2; Supplementary Table S3). Interestingly, the entire LPE O-class, including 16:1; 18:1 and 18:2, was significantly reduced in both PCSK9 KO brain areas (Figures 3E–I, 4; Tables 1, 2; Supplementary Tables S2, S3). While the changes in LPA, LPE, PC, PC O-and PI lipid classes were not statistically significant (Figures 3E,H; Table 2), the levels of several individual species differed significantly between genotypes (Figure 4; Supplementary Table S3). For example, LPA 18:0, LPE 18:0 and PI 36:2 were decreased up to 37%, whereas several PC species (PC 38:6, 40:6, 42:1, 42:2) increased up to 35%, and PC O-species (PC O-40:1, PC O-42:4, PC O-42:6) increased up to 54% in the KO cerebral cortex (Figure 4; Supplementary Table S3). In addition, we observed a 19% enrichment of SL class HexCer, including almost all detected species (Figures 3E,H, 4; Table 2; Supplementary Table S3) in PCSK9 KO cerebral cortex. Even though there were no changes in the class levels of the remaining SL, several species of the various SL classes (Cer 42:2;2, SHexCer 42:1;2 and SM 42:2;2) had increased by up to 26% or alternatively decreased (monosialodihexosylganglioside (GM3) 42:2;2) by 35% in the KO cerebral cortex (Figure 4; Supplementary Table S3). Interestingly, very long chain fatty acid (VLCFA) containing species 42:1;2 of HexCer and SHexCer were increased in the PCSK9 KO brain in both cerebral cortex and cerebellum (Figure 4; Supplementary Tables S2, S3). Finally, no significant change in cholesterol levels was observed in PCSK9 KO cerebral cortex (Figures 3D,E; Table 2).

### 3.4. PCSK9 knockout mice display unchanged cognition and motor learning

**Figure 5:**
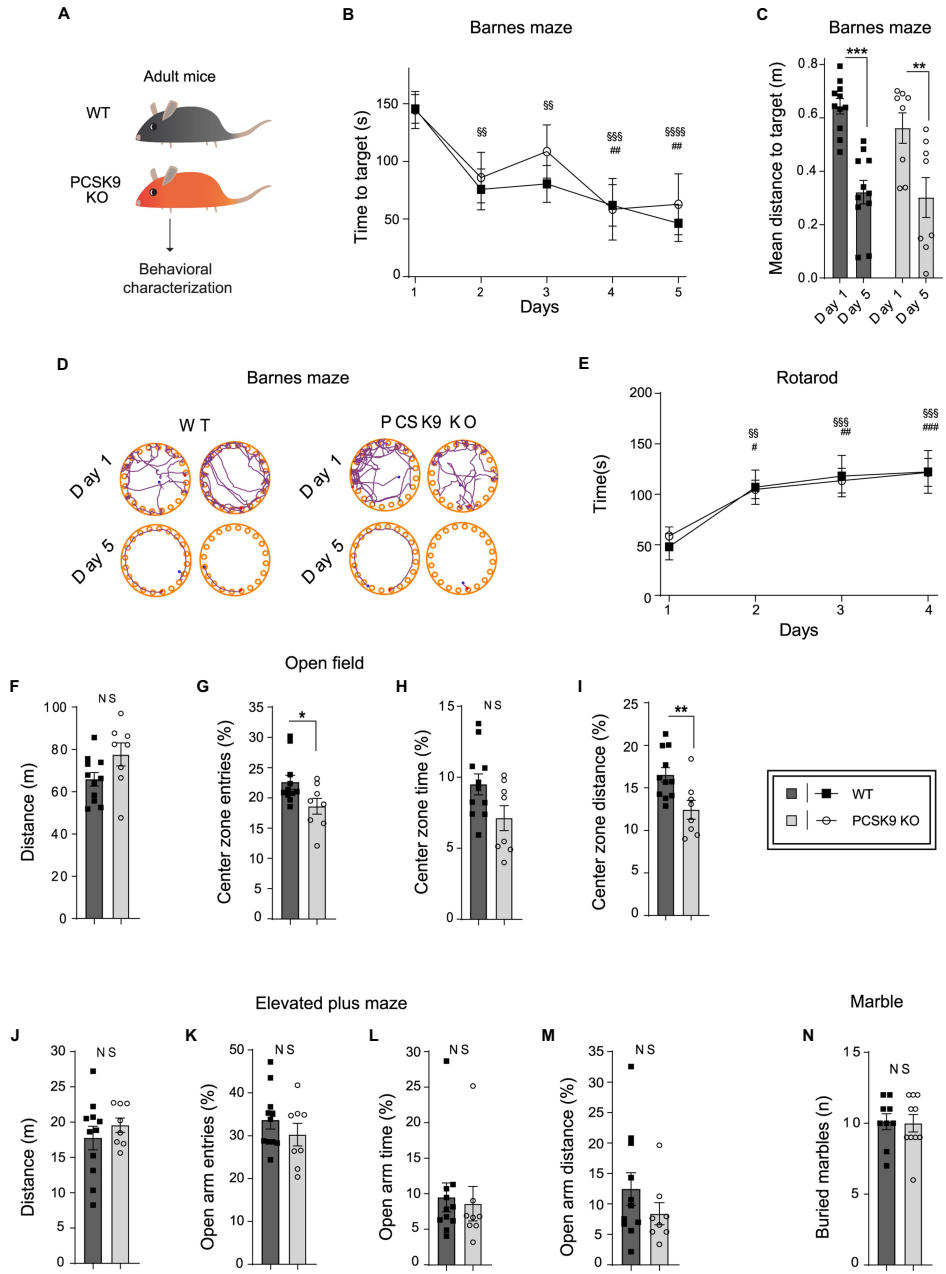
Behavioral characterization of PCSK9 KO mice. **(A)** Schematic representation of the experiment. **(B–D)** Barnes maze. Time to reach target **(B)**. Mean distance to target **(C)**. Representative examples of search patterns at Day 1 and Day 5 **(D)** (*N* = 11 WT and 8 KO mice per group). **(E)** Rotarod.(Continued)FIGURE 5 (Continued)Time spent on the rod (*n* = 9 WT and 10 KO mice per group). **(F–I)** Open field test. Distance travelled **(F)**. Entries to the center zone **(G)**. Time spent in the center zone **(H)**. Distance travelled in the center zone **(I)** (*N* = 11 WT and 8 KO mice per group). **(J–M)** Elevated plus maze. Distance travelled **(J)**. Open arm entries **(K)**. Time spent in open arms **(L)**. Distance travelled in open arms **(M)** (*N* = 11 WT and 8 KO mice per group). **(N)** Marble burying test. Number of buried marbles (*n* = 9 WT and 10 KO mice per group). For all behavioral tests, 9-to-11-week-old male mice were used. All data are represented as mean ± SEM. Statistical significance was evaluated using a two-tailed Student’s *t*-test (**C**, **F–N**). Data of panels **B,E** are analyzed using two-way ANOVA RM. **(B)** Interaction *F*(4,68) = 0.4; *p* > 0.8; Main effect time. **(E)** Interaction *F*(3, 51) = 0.20; *p* > 0.89. Main effect time. Sidak’s multiple comparisons adjusted *p* values WT §, KO #. Asterisks indicate significantly different values (NS *p* > 0.05; *p ≤ 0.05; **p ≤ 0.01; ***p ≤ 0.001).

To investigate the impact of PCSK9 deficiency on brain function, we studied PCSK9-deficient mice and WT controls in a battery of behavioral tests assessing spatial-and motor learning as well as anxiety-related behavior (Figure 5). First, the mice were tested in Barnes maze, which measures their ability to apply spatial learning and memory to find an escape-hole on a round table with 20 identical holes. No difference was observed between the genotypes with regard to learning and recollection of the correct escape hole over time (Figures 5B–D), suggesting normal spatial memory function in the absence of PCSK9. Similarly, no defects in motor learning abilities were observed in an accelerating rotarod test for PCSK9 KO mice compared to WT controls (Figure 5E). PCSK9 KO mice also displayed normal motor activity during 20 min in the open field test (Figures 5F–I). The KO mice entered and travelled slightly less in the center zone of the open field arena (Figures 5G,I), indicating a potential increase in general anxiety levels. However, no behavioral change was observed in an elevated plus maze (Figures 5J–M), which is a more specific test for general anxiety levels in mice. We finally monitored the behavior associated with burying colorful marbles (Figure 5N). Burying interesting objects is normal mouse behavior, but excessive burying could be interpreted as a compulsive or anxious behavior (de Brouwer et al., 2019). However, no difference between genotypes was observed.

## 4. Discussion

The brain consists of 60% lipids with cholesterol being the most abundant, rendering it the tissue with the highest cholesterol content in the body. Cholesterol metabolism plays a key role in the CNS both during early development and adulthood. It is an essential component of the plasma membrane and therefore critical for myelin sheath formation and synaptic plasticity. Accordingly, defects in CNS cholesterol metabolism are strongly associated with neurological disorders including Alzheimer’s disease. However, it remains unclear how changes in plasma cholesterol might affect CNS sterol metabolism and if such changes affect cognitive function, and no evidence suggest a net transfer of sterols from the periphery to the CNS (Dietschy and Turley, 2004; Chang et al., 2017). Our present study shed light on this question by addressing how the potent influence of PCSK9 on plasma cholesterol could regulate any potential sterol or lipid exchange with the CNS thereby affecting its overall lipid composition. Furthermore, we also address how locally expressed PCSK9 might impact brain lipid composition directly, and if PCSK9 affects neuronal function through interactions with receptor systems, independently of its role in cholesterol metabolism.

Several studies have reported that PCSK9 affects peripheral lipids beyond cholesterol and hence plays a key role in systemic lipid metabolism (Jänis et al., 2013; Hilvo et al., 2018). However, lipids also carry out various important roles in the brain. They regulate geometric properties of neuronal and glial membranes and act as direct ligands for proteins (Piomelli et al., 2007). Membrane lipids also play important roles in regulating ion channels (Korinek et al., 2015), lysosomal function (Schulze and Sandhoff, 2011), apoptosis and non-apoptotic cell death (Fadok et al., 1992; Magtanong et al., 2016). To understand the consequences of lifelong PCSK9 deficiency on brain lipid composition, we characterized the lipidome including cholesterol of cerebellum and cortex from PCSK9 KO mice and WT controls using shotgun lipidomics, and found changes in various lipid classes and species of these brain areas (Figures 3, 4; Tables 1, 2; Supplementary Tables S2, S3). Cholesterol levels were unchanged in cortex but a small albeit significant change was observed in the KO cerebellum (Figures 3D–F; Tables 1, 2). Thus, the low plasma cholesterol in the PCSK9 KO mice (Rashid et al., 2005) does not appear to markedly impact brain cholesterol levels, supporting the hypothesis that peripheral and CNS cholesterol metabolism operate independently of each other (Dietschy and Turley, 2004; Chang et al., 2017). The most notable changes in lipid classes were observed for cerebellum content of GPLs including PA which is the precursor for all neural membrane GPLs and could affect the metabolism of several GPL (Figures 3E,F, 4; Table 1; Supplementary Table S2). GPLs are one of the major groups of membrane lipids and play key roles in membrane fluidity and cellular signaling (Farooqui et al., 2000). Of note, the GPL classes PE and PC that both had changed in the PCSK9 KO cerebellum by over 40% (Figures 3E,F; Table 1) are known as key regulators of membrane fluidity besides cholesterol (Dawaliby et al., 2016). The cellular signaling lipid LPA was reduced by 28% in the PCSK9 KO cerebellum compared to WT (Figures 3E,F; Table 1; Moolenaar, 1995). The SL class of membrane lipids including ganglioside GM1 and sulfatide (SHexCer) were also significantly altered in the adult PCSK9 KO mouse cerebellum (Figures 3E,F; Table 1). In addition to the changes in overall class levels, PCSK9 deficiency affected the acyl chain length and the degree of unsaturation of various PC and SL species in cerebellum (Figures 3G, 4; Supplementary Table S2). Those properties determine the specific role and position of the lipid in the lipid membrane and thus also the functional efficacy of the neural membranes. The lipid changes in PCSK9 KO cortex were less pronounced (Figures 3, 4; Table 2; Supplementary Table S3) and did not correlate with those of the cerebellum with the exception of LPE O-and VLCFA 42:1;2 containing species of HexCer and SHexCer (Figures 3E,F,H, Tables 1, 2; Supplementary Tables S2, S3).

At the functional level, PCSK9 KO mice had intact memory function and displayed normal behavior across a range of tests including cerebellum dependent motor learning (Figure 5). This clearly suggests that the observed lipid changes in the absence of PCSK9 did not translate into behavioral deficits, although we cannot exclude that an extended analysis of phenotypes specifically related to the cerebellum might reveal subtle phenotypes overlooked in the present study. Our findings are in agreement with a recent study that evaluated the effect of an anti-PCSK9 antibody treatment on cognitive function, locomotion and anxiety of two different mouse strains and found no evidence of adverse effects caused by PCSK9 inhibition (Schlunk et al., 2021). We speculate that the observed lipidomic changes in PCSK9 KO cortex and cerebellum could be an adaptation mechanism to secure membrane properties such as membrane curvature, bending, fluidity and interactions with neighboring lipids and proteins in response to the reduced levels of lipoprotein particles in the periphery. However, this only applies to lifelong PCSK9 deficiency and we cannot conclude if similar adaptive changes will occur when PCSK9 deficiency is pharmacologicaly induced later on in life. Of note, the observed lipidomic profiles of PCSK9 KO cerebellum or cortex share no similarities with the reported lipidomic profiles of the plasma from PCSK9-deficient mice or from humans carrying PCSK9 loss-of-function variant R46L (Jänis et al., 2013), likely reflecting that lipid metabolism in the CNS and periphery is fundamentally different. Despite the absence of a cognitive phenotype in PCSK9 KO mice, we did observe a direct effect of purified PCSK9 on LDLR levels and neurite complexity of developing neurons in culture (Figures 1C–F), demonstrating that the LDLR degradation machinery indeed is present. Accordingly, previous studies reported increased levels of LDLR in embryonic telencephalon of PCSK9 KO mice (Rousselet et al., 2011), and that PCSK9 promoted neuronal differentiation in an *in vitro* model of telencephalon neurons (Seidah et al., 2003). However, we observed no significant difference in LDLR levels in WT and PCSK9 KO cerebellum homogenates from adult mice (Figure 1G) which is in line with previously reported observations in cortex, hippocampus and olfactory bulb of adult PCSK9 KO mice compared to WT (Liu et al., 2010; Rousselet et al., 2011).

In the brain, PCSK9 expression correlates with expression of LDLR-related receptor VLDLR and the adaptor protein Disabled 1 (Dab1)^4^, which both, together with ApoER2, are components of the Reelin signaling pathway and hence play a central role in neuronal migration and proper cortical layering (Sekine et al., 2014). Interestingly, PCSK9 has been reported to induce degradation and modulate the function of VLDLR and ApoER2 *in vitro* (Poirier et al., 2008; Kysenius et al., 2012) while knockdown of PCSK9 mRNA in zebrafish results in highly aberrant neuronal development and embryonic death (Poirier et al., 2006). However, we observed that neither knockout nor overexpression of PCSK9 in the developing cortex at the critical period of laminar development interfered with the cortical development process (Figure 2; Supplementary Figure S2; Supplementary Tables S1A–D), indicating that PCSK9 has no major role in regulation of Reelin receptor levels at this developmental stage.

Taken together, our findings demonstrate that lack of PCSK9 during development and until adulthood does not have adverse impact on brain function in male mice. However, it is possible that PCSK9-related phenotypes may emerge in aging mice and could be dependent on the genetic background. Our findings are in line with data from PCSK9 inhibitor clinical trials (Giugliano et al., 2017; Mannarino et al., 2018; Gencer et al., 2020; Ray et al., 2020) and from observations that people carrying loss-of-function mutations of the PCSK9 gene have no signs of neurological defects (Cohen et al., 2005, 2006; Zhao et al., 2006; Hooper et al., 2007; Mefford et al., 2018).

## Data availability statement

The original contributions presented in the study are included in the article/Supplementary material, further inquiries can be directed to the corresponding authors.

## Ethics statement

The animal study was reviewed and approved by Danish Animal Experiments Inspectorate (2016-15-0201-01127); Landesamt für Gesundheit und Soziales (LaGeSo), permission number G0054/19.

## Author contributions

AP, PM, MA, RG, TT, VT, CG, and SG: conceptualization. AP, MK, EB, MB, JV, PM, RG, and CG: methodology. AP, JT, MK, and ME: formal analysis. AP, DO, MK, EB, ME, and MB: investigation. VT, CG, and SG: resources. AP, TT, VT, and SG: funding acquisition. MA, TT, VT, CG, and SG: supervision. AP, VT, CG, and SG: project administration. AP and JT: data curation. DO and JT: validation. AP, DO, JT, and EB: visualization. AP: writing original draft. DO, JT, MK, MB, TT, CG, and SG: writing–review and editing. All authors contributed to the article and approved the submitted version.

## Funding

This study was funded by the Lundbeck Foundation (SG), Novo Nordisk Foundation (SG), Carlsberg Foundation (SG), Kristjan Jaak Scholarship program “Degree Studies Abroad” of Foundation Archimedes” (AP), Danish Council for Independent Research Sapere Aude starting grant (SG, grant number DFF 4183–00604). TT and JT were supported by Estonian Research Council (grant PRG805), European Union through the European Regional Development Fund (Project No. 2014–2020.4.01.15–0012), H2020-MSCA-RISE-2016 (EU734791), and Protobios grant (5.1–4/20/170) from the Estonian Ministry of Education. IUE experiments were funded by Russian Science Foundation, grant number 21–65-00017 (VT and EB).

## Conflict of interest

JV, PM, SG, and CG have significant financial interests in Draupnir Bio, a company involved in the development of small molecule inhibitors of PCSK9. JT and TT are employed by Protobios LLC. JT is employed by Dxlabs LLC.

The remaining authors declare that the research was conducted in the absence of any commercial or financial relationships that could be construed as a potential conflict of interest.

## Publisher’s note

All claims expressed in this article are solely those of the authors and do not necessarily represent those of their affiliated organizations, or those of the publisher, the editors and the reviewers. Any product that may be evaluated in this article, or claim that may be made by its manufacturer, is not guaranteed or endorsed by the publisher.

## Footnotes

^1^ https://github.com/ELELAB/lipidQ

^2^ https://biit.cs.ut.ee/clustvis/

^3^ https://github.com/taunometsalu/ClustVis

^4^ proteinatlas.org

## Abbreviations

APLP2: amyloid precursor-like protein 2
ApoER2: apolipoprotein receptor type 2
APP: amyloid precursor protein
Cer: ceramide
DAG: diacylglycerol
FC: cholesterol
GM1: monosialotetrahexosylganglioside
GM3: monosialodihexosylganglioside
HexCer: hexosyl ceramide (GlcCer or GalCer)
HSPG: heparan sulfate proteoglycans
IUE: *in utero* electroporation
LPC: lysophosphatidylcholine
LPE: lysophosphatidylethanolamine
LPG: lysophosphatidylglycerol
LPI: lysophosphatidylinositol
LPS: lysophosphatidylserine
LPA: lysophosphatic acid
NARC1: neural apoptosis-regulated convertase-1
PA: phosphatidic acid
PC: phosphatidyl choline
PCSK9: proprotein convertase subtilisin/kexin type 9
PE: phosphatidyl ethanolamine
PG: phosphatidylglycerol
PI: phosphatidylinositol
PS: phosphatidylserine
SM: sphingomyelin
SHexCer: sulfatide
VLDLR: very-low density lipoprotein receptor
