# Supplementary material for: PCSK9 deficiency alters brain lipid composition without affecting brain development and function
Source: Front Mol Neurosci. 2023 Jan 17;15:1084633. doi: 10.3389/fnmol.2022.1084633 (PMC9887304; doi:10.3389/fnmol.2022.1084633)
Supplement: Supplementary file 1 [file Data_Sheet_1.PDF]

Supplementary Material

1 Supplementary Figures

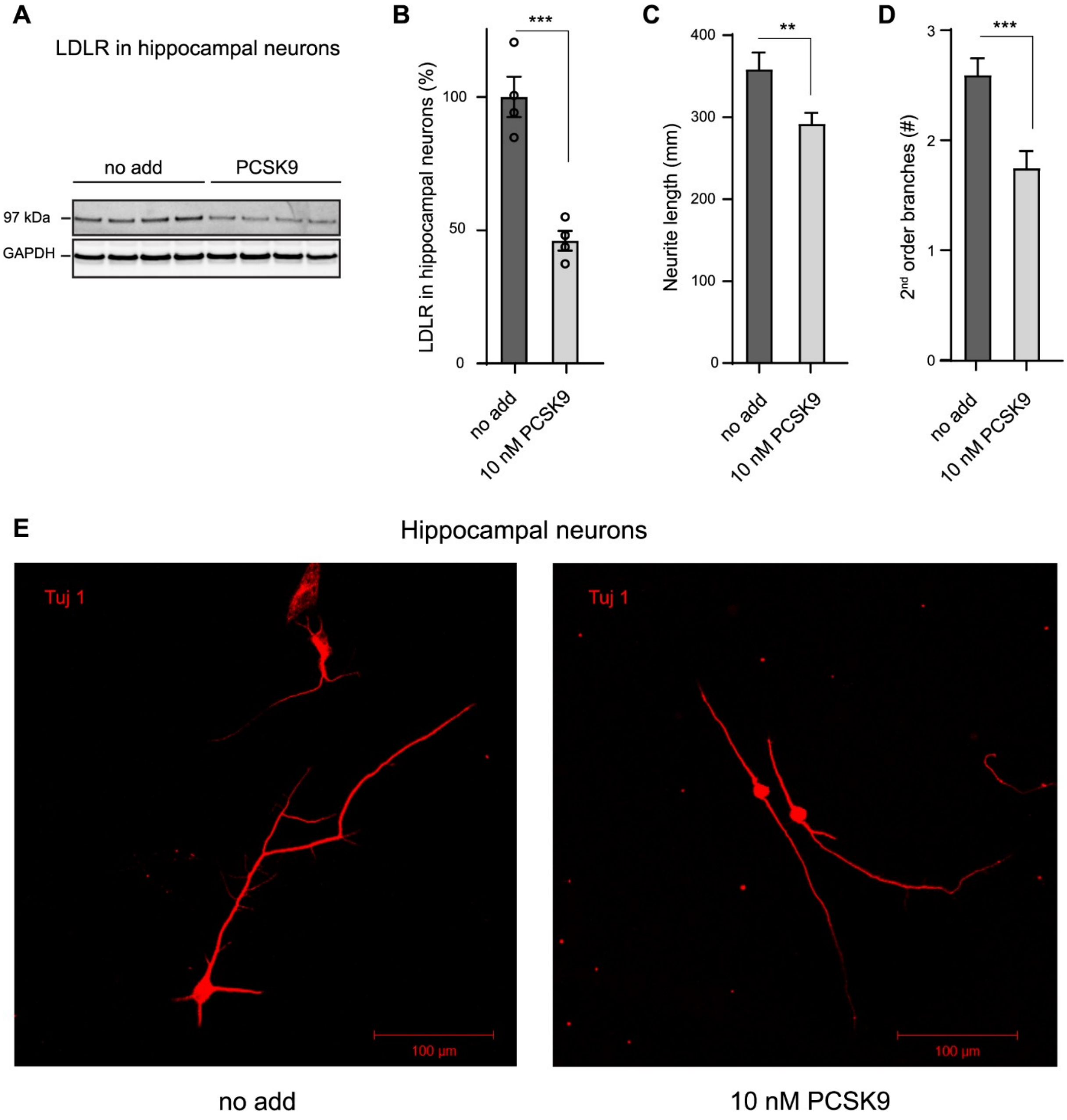

Supplementary Figure S1. PCSK9 targets LDLR and reduces neurite length and branching when added to hippocampal neurons in culture. Representative Western blot of LDLR receptor in homogenates of hippocampal neurons (A). Primary cultures were supplemented with PCSK9 (10 nM) at 1 days in vitro (DIV) and lysed at 3 DIV. Densitometric quantification of LDLR normalized to GAPDH (n = 4) (B). Neurite length (n=98-100) (C) and branching (n=55-71) (D) of cells, analyzed following immunostaining for the neuronal marker Tuj1 (E). Data are represented as mean

± SEM. Statistical significance was evaluated using a two-tailed Student's t-test (** $P \leq 0.01$; *** $P \leq 0.001$).

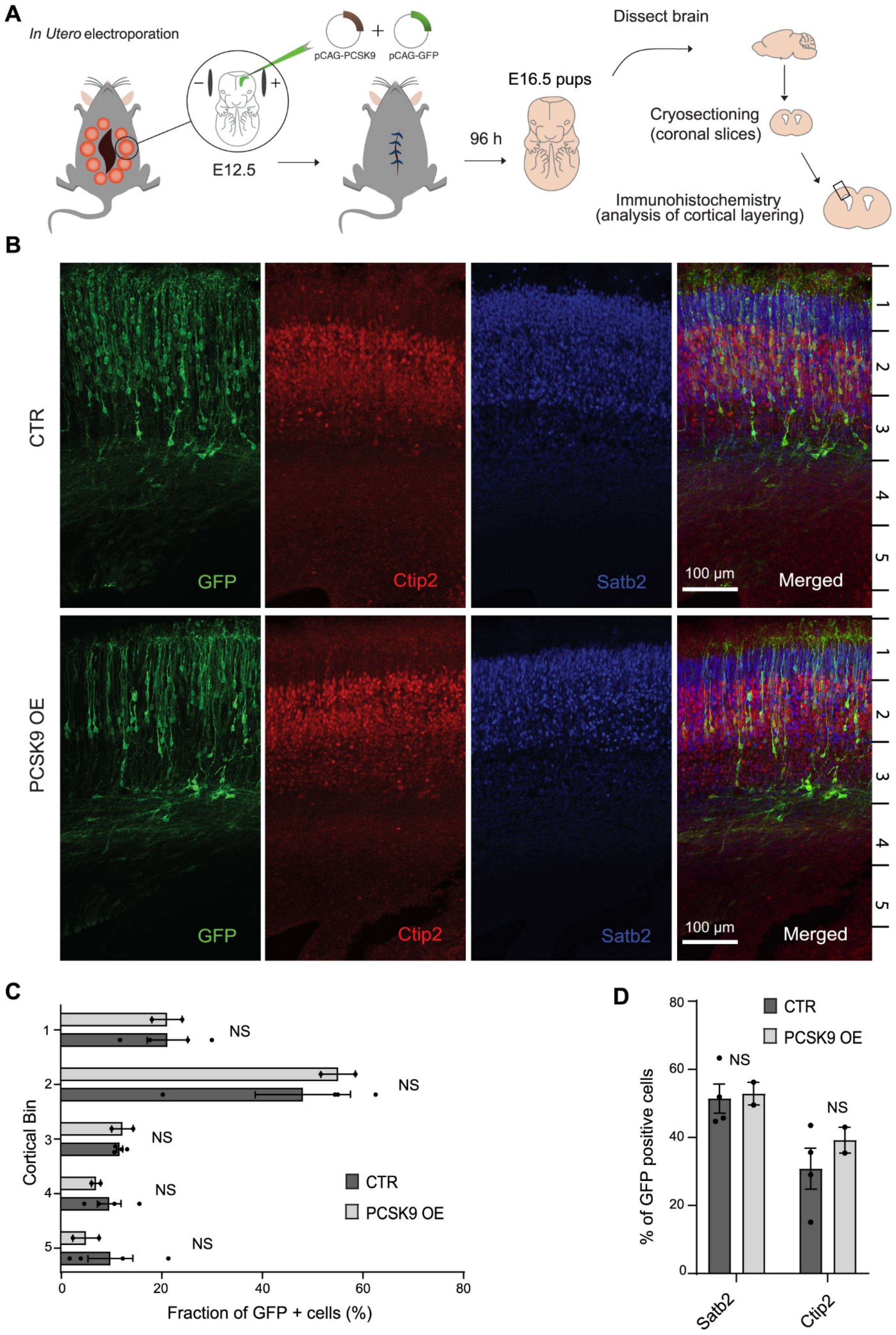
A
In Utero electroporation
pCAG-PCSK9
pCAG-GFP
E12.5
96 h
E16.5 pups
Dissect brain
Cryosectioning
(coronal slices)
Immunohistochemistry
(analysis of cortical layering)
B
CTR
PCSK9 OE
GFP
Ctip2
Satb2
100 μm
Merged
1
2
3
4
5
C
Cortical Bin
Fraction of GFP + cells (%)
NS
CTR
PCSK9 OE
0
20
40
60
80
D
% of GFP positive cells
CTR
PCSK9 OE
NS
Satb2
Ctip2

Supplementary Figure S2. PCSK9 overexpression during embryonic development has no effect on cortical layering. (A) Schematic representation of the IUE experiment. (B) Representative images of immunostaining of E16.5 cortices *in utero* electroporated with pCAG-empty and pCAG-GFP plasmid (CTR) or pCAG-PCSK9 and pCAG-GFP plasmid (PCSK9 OE) at E12.5. (C) Quantification of laminar distribution of cortical neurons labelled with GFP in E16.5 cortices *in utero* electroporated with pCAG-empty and pCAG-GFP plasmid (CTR) or pCAG-PCSK9 and pCAG-GFP plasmid (PCSK9 OE) at E12.5 (See also Table S1C). Cortical bin - the maximum height of the cortical slice was divided into 5 bins of identical dimensions. (D) Quantification of the fraction of GFP positive neurons expressing indicated fate markers (Satb2, Ctip2) in E16.5 cortices electroporated with pCAG-empty and pCAG-GFP plasmid (CTR) or pCAG-PCSK9 and pCAG-GFP plasmid (PCSK9 OE) at E12.5 (See also Table S1D). Results on graphs are represented as average percent ± SEM. For statistical analyses two-way ANOVA with Bonferroni multiple comparison test was performed, alpha = 0.05. All p values were above 0.05 and considered non-significant; n = 4 CTR, 2 OE.

2 Supplementary Tables

2.1 Supplementary Tables S1A-B. Quantifications of neuronal fate markers in WT and PCSK9 KO embryonic brain

2.1.1 Supplementary Table S1A

	WT	PCSK9 KO	p value
% Satb2+ Neurons	34.47 ± 7.85	40.00 ± 4.14	$p > 0.05$
% Ctip2+ Neurons	11.2 ± 2.44	13.53 ± 1.44	$p > 0.05$
number of brains	3	3	

Quantification of the fraction of all neurons (stained with DRAQ5 nuclear marker) in WT or PCSK9 KO mouse brain at E17.5, positive for respective fate markers (Satb2 or Ctip2). Indicated is average fraction ± S.D. Numbers of analyzed brains are at the bottom row of the table. Statistical analysis: two-way ANOVA with Bonferroni multiple comparison test.

2.1.2 Supplementary Table S1B

Cortical bin	WT Satb2	PCSK9 KO Satb2	WT Ctip2	PCSK9 KO Ctip2	p value
1	36.50 ± 0.78	31.91 ± 8.29	29.40 ± 7.87	20.87 ± 18.39	$p > 0.05$
2	25.50 ± 7.18	25.52 ± 3.55	56.61 ± 15.04	55.16 ± 5.69	$p > 0.05$
3	24.56 ± 3.58	20.34 ± 1.36	13.25 ± 7.66	21.88 ± 13.18	$p > 0.05$
4	12.95 ± 8.85	21.01 ± 8.97	0.12 ± 0.21	0.70 ± 0.88	$p > 0.05$
5	0.50 ± 0.87	1.22 ± 1.80	0.62 ± 1.07	1.39 ± 1.45	$p > 0.05$
number of brains	3	3	3	3	

Quantification of laminar distribution of Satb2 or Ctip2 positive cortical neurons in WT or in PCSK9 KO mouse brains at E17.5. Indicated are average fractions of neurons in each cortical bin ± SD. Numbers of analyzed brains are at the bottom row of the table. Statistical analysis: two-way ANOVA with Bonferroni multiple comparison test.

2.2 Supplementary tables S1C-D. Quantifications of neuronal fate markers in WT and PCSK9 OE embryonic brain

2.2.1 Supplementary Table S1C

Cortical bin	CTR (pCAG, GFP, Venus)	OE (PCSK9, GFP, Venus)	p value
1	21.10 ± 8.07	21.01 ± 4.26	$p > 0.05$
2	48.03 ± 18.93	55.06 ± 4.86	$p > 0.05$
3	11.60 ± 1.18	12.14 ± 3.03	$p > 0.05$
4	9.52 ± 4.70	6.90 ± 1.27	$p > 0.05$
5	9.75 ± 8.94	4.89 ± 3.70	$p > 0.05$
number of brains	4	2	

Quantification of laminar distribution of cortical neurons in E16.5 WT mouse brains after electroporation with indicated vectors at E12.5. Indicated are average fractions of transfected neurons in each cortical bin ± S.D. Numbers of analyzed brains are at the bottom row of the table. CTR – control, OE – PCSK9 overexpression. Statistical analysis: two-way ANOVA with Bonferroni multiple comparison test between all pairs.

2.2.2 Supplementary Table S1D

	CTR (pCAG, GFP, Venus)	OE (PCSK9, GFP, Venus)	p value
% GFP+ Satb2+ Neurons	51.4 ± 8.55	52.85 ± 4.74	$p > 0.05$
% GFP+ Ctip2+ Neurons	30.80 ± 12.03	39.20 ± 5.37	$p > 0.05$
number of brains	4	2	

Quantification of the fraction of GFP+ neurons expressing indicated vectors, positive for respective fate markers (Satb2 or Ctip2). Indicated is average fraction ± S.D. Numbers of analyzed brains are at the bottom row of the table. CTR – control, OE – PCSK9 overexpression. Statistical analysis: two-way ANOVA with Bonferroni multiple comparison test.

2.2.3 Supplementary Table S2. Lipid species detected in WT and KO cerebellum

	Average mol% ± SEM						
	WT			PCSK9 KO			p value
FC	58.806	±	0.472	57.325	±	0.515	0.0499
DAG 30:0	0.012	±	0.009	0.008	±	0.002	0.6444
DAG 30:1	0.005	±	0.003	0.007	±	0.002	0.5004
DAG 32:0	0.015	±	0.006	0.023	±	0.004	0.3066
DAG 32:1	0.009	±	0.004	0.017	±	0.005	0.2209
DAG 34:0	0.012	±	0.007	0.011	±	0.002	0.9752
DAG 34:1	0.148	±	0.009	0.135	±	0.007	0.2832
DAG 34:2	0.012	±	0.002	0.019	±	0.005	0.1943
DAG 36:1	0.123	±	0.008	0.108	±	0.005	0.1334
DAG 36:2	0.062	±	0.003	0.059	±	0.005	0.6581
DAG 36:3	0.004	±	0.001	0.012	±	0.004	0.0838
DAG 36:4	0.043	±	0.002	0.039	±	0.003	0.3760
DAG 38:1	0.031	±	0.006	0.032	±	0.006	0.9038
DAG 38:2	0.023	±	0.002	0.018	±	0.001	0.0198
DAG 38:3	0.002	±	0.000	0.003	±	0.001	0.6105
DAG 38:4	0.168	±	0.009	0.146	±	0.008	0.0874
DAG 38:5	0.026	±	0.002	0.021	±	0.001	0.0368
DAG 38:6	0.025	±	0.002	0.023	±	0.001	0.3483
DAG 40:1	0.019	±	0.005	0.023	±	0.006	0.6244
DAG 40:2	0.008	±	0.001	0.007	±	0.001	0.4376
DAG 40:4	0.012	±	0.001	0.010	±	0.000	0.0234
DAG 40:6	0.020	±	0.001	0.021	±	0.001	0.8220
DAG 42:1	0.008	±	0.002	0.009	±	0.001	0.7771
DAG 42:2	0.005	±	0.001	0.005	±	0.001	0.8044
Cer 34:1;2	0.012	±	0.007	0.016	±	0.004	0.6578
Cer 36:1;2	0.124	±	0.006	0.131	±	0.005	0.3998
Cer 36:2;2	0.014	±	0.003	0.017	±	0.003	0.4306
Cer 38:1;2	0.027	±	0.004	0.032	±	0.003	0.3903
Cer 40:1;2	0.008	±	0.005	0.010	±	0.003	0.7594
Cer 40:2;2	0.013	±	0.002	0.015	±	0.002	0.6133
Cer 42:1;2	0.014	±	0.004	0.014	±	0.004	0.9015
Cer 42:2;2	0.041	±	0.002	0.042	±	0.002	0.7660

GM1 36:1;2	0.218	±	0.006	0.195	±	0.006	0.0124
GM3 34:1;2	0.002	±	0.000	0.002	±	0.000	0.9360
GM3 36:1;2	0.053	±	0.002	0.054	±	0.001	0.5009
GM3 38:1;2	0.014	±	0.001	0.012	±	0.001	0.0954
GM3 40:1;2	0.004	±	0.000	0.003	±	0.000	0.3027
GM3 40:2;2	0.004	±	0.000	0.003	±	0.000	0.0934
HexCer 36:1;2	0.147	±	0.006	0.130	±	0.004	0.0266
HexCer 36:2;2	0.004	±	0.001	0.003	±	0.000	0.2005
HexCer 38:1;2	0.033	±	0.003	0.034	±	0.003	0.8046
HexCer 40:1;2	0.277	±	0.010	0.242	±	0.006	0.0111
HexCer 40:2;2	0.221	±	0.010	0.178	±	0.005	0.0014
HexCer 42:1;2	0.467	±	0.013	0.504	±	0.010	0.0370
HexCer 42:2;2	2.213	±	0.086	2.062	±	0.057	0.1636
HexCer 44:0;2	0.056	±	0.010	0.066	±	0.008	0.4662
HexCer 44:2;2	0.009	±	0.001	0.011	±	0.001	0.2513
SHexCer 36:1;2	0.011	±	0.002	0.007	±	0.001	0.1558
SHexCer 40:0;2	0.022	±	0.002	0.033	±	0.002	0.0011
SHexCer 42:1;2	0.043	±	0.005	0.077	±	0.006	0.0004
SHexCer 42:2;2	0.336	±	0.027	0.422	±	0.026	0.0374
SM 34:1;2	0.081	±	0.002	0.082	±	0.002	0.8220
SM 36:1;2	1.552	±	0.020	1.575	±	0.028	0.5112
SM 36:2;2	0.035	±	0.001	0.036	±	0.001	0.9005
SM 38:2;2	0.003	±	0.000	0.003	±	0.000	0.4806
SM 40:1;2	0.075	±	0.003	0.072	±	0.002	0.4027
SM 40:2;2	0.025	±	0.002	0.019	±	0.003	0.1272
SM 42:1;2	0.008	±	0.001	0.012	±	0.001	0.0272
SM 42:2;2	0.373	±	0.018	0.348	±	0.007	0.2173
LPA 16:0	0.011	±	0.001	0.009	±	0.001	0.0378
LPA 18:0	0.004	±	0.001	0.004	±	0.001	0.9783
LPA 18:1	0.018	±	0.002	0.013	±	0.001	0.0043
LPC 16:0	0.170	±	0.015	0.142	±	0.012	0.1555
LPC 18:0	0.085	±	0.006	0.071	±	0.005	0.1112
LPC 18:1	0.064	±	0.004	0.057	±	0.004	0.2328
LPC 18:3	0.013	±	0.004	0.009	±	0.001	0.3454
LPC 20:1	0.005	±	0.001	0.004	±	0.001	0.1526

LPC 20:3	0.006	±	0.002	0.005	±	0.000	0.4602
LPC 20:4	0.006	±	0.002	0.006	±	0.000	0.7665
LPC 22:6	0.014	±	0.001	0.014	±	0.001	0.4208
LPE 16:0	0.055	±	0.006	0.047	±	0.002	0.2277
LPE 16:1	0.012	±	0.001	0.011	±	0.001	0.5655
LPE 18:0	0.065	±	0.007	0.056	±	0.002	0.2367
LPE 18:1	0.423	±	0.052	0.371	±	0.023	0.3741
LPE 18:2	0.008	±	0.001	0.007	±	0.000	0.5997
LPE 20:4	0.150	±	0.018	0.127	±	0.011	0.2700
LPE 22:4	0.086	±	0.011	0.069	±	0.006	0.2133
LPE 22:5	0.006	±	0.000	0.004	±	0.000	0.0792
LPE 22:6	0.347	±	0.038	0.335	±	0.027	0.8089
LPE O-16:1	0.010	±	0.001	0.008	±	0.000	0.0299
LPE O-18:1	0.017	±	0.001	0.014	±	0.000	0.0294
LPE O-18:2	0.009	±	0.001	0.007	±	0.000	0.0032
LPI 16:0	0.007	±	0.001	0.006	±	0.001	0.4588
LPI 18:0	0.028	±	0.002	0.025	±	0.002	0.3068
LPI 18:1	0.003	±	0.001	0.003	±	0.001	0.7887
LPS 18:0	0.103	±	0.012	0.085	±	0.008	0.2346
LPS 18:1	0.031	±	0.003	0.025	±	0.002	0.0484
PA 32:0	0.002	±	0.000	0.003	±	0.000	0.0020
PA 34:1	0.088	±	0.004	0.095	±	0.005	0.2722
PA 36:1	0.054	±	0.003	0.061	±	0.004	0.1169
PA 36:2	0.039	±	0.002	0.044	±	0.002	0.0808
PA 36:4	0.006	±	0.001	0.012	±	0.002	0.0061
PA 38:2	0.006	±	0.001	0.007	±	0.001	0.9085
PA 38:4	0.021	±	0.001	0.022	±	0.001	0.4780
PA 38:5	0.003	±	0.000	0.003	±	0.000	0.9872
PA 40:6	0.004	±	0.001	0.006	±	0.001	0.0799
PA 44:4	0.007	±	0.002	0.027	±	0.005	0.0015
PA O-36:2	0.002	±	0.000	0.004	±	0.001	0.0904
PC 30:0	0.073	±	0.002	0.069	±	0.001	0.0416
PC 32:0	3.265	±	0.053	3.250	±	0.024	0.7955
PC 32:1	0.255	±	0.005	0.245	±	0.003	0.1109
PC 34:0	0.961	±	0.014	0.951	±	0.014	0.5972

PC 34:1	8.656	±	0.130	8.437	±	0.097	0.1944
PC 34:2	0.315	±	0.003	0.325	±	0.006	0.1313
PC 34:3	0.151	±	0.009	0.146	±	0.003	0.5857
PC 34:4	0.008	±	0.001	0.008	±	0.000	0.9496
PC 36:1	3.192	±	0.068	2.984	±	0.044	0.0210
PC 36:2	0.852	±	0.014	0.805	±	0.014	0.0304
PC 36:3	0.135	±	0.004	0.161	±	0.011	0.0451
PC 36:4	1.053	±	0.036	1.005	±	0.025	0.2990
PC 36:5	0.027	±	0.001	0.029	±	0.002	0.4473
PC 38:1	0.266	±	0.008	0.227	±	0.004	0.0004
PC 38:2	0.206	±	0.006	0.174	±	0.004	0.0005
PC 38:3	0.017	±	0.004	0.048	±	0.016	0.0759
PC 38:4	0.885	±	0.027	0.844	±	0.022	0.2473
PC 38:5	0.204	±	0.008	0.194	±	0.006	0.3091
PC 38:6	1.445	±	0.088	1.489	±	0.061	0.6836
PC 40:1	0.058	±	0.003	0.053	±	0.001	0.0941
PC 40:2	0.057	±	0.002	0.048	±	0.001	0.0061
PC 40:4	0.082	±	0.002	0.076	±	0.002	0.0332
PC 40:6	1.658	±	0.077	1.660	±	0.058	0.9853
PC 40:7	0.378	±	0.024	0.372	±	0.016	0.8317
PC 40:8	0.063	±	0.013	0.060	±	0.010	0.8575
PC 40:9	0.052	±	0.005	0.050	±	0.003	0.7848
PC 42:1	0.057	±	0.002	0.057	±	0.001	0.8173
PC 42:2	0.050	±	0.002	0.047	±	0.001	0.1979
PC 42:5	0.002	±	0.000	0.002	±	0.000	0.7569
PC 42:7	0.065	±	0.004	0.059	±	0.002	0.1773
PC 42:8	0.003	±	0.000	0.003	±	0.001	0.8655
PC 42:9	0.053	±	0.005	0.052	±	0.003	0.8449
PC 44:10	0.006	±	0.001	0.005	±	0.001	0.5905
PC 44:2	0.005	±	0.001	0.005	±	0.001	0.9865
PC O-32:0	0.011	±	0.001	0.010	±	0.000	0.1189
PC O-32:1	0.004	±	0.000	0.003	±	0.000	0.3444
PC O-32:2	0.008	±	0.001	0.008	±	0.000	0.4292
PC O-34:0	0.011	±	0.001	0.010	±	0.001	0.4879
PC O-34:1	0.088	±	0.003	0.076	±	0.001	0.0015

PC O-34:2	0.020	±	0.001	0.018	±	0.001	0.2974
PC O-34:3	0.006	±	0.001	0.006	±	0.003	0.8655
PC O-36:1	0.028	±	0.001	0.022	±	0.001	0.0043
PC O-36:2	0.018	±	0.003	0.020	±	0.001	0.6748
PC O-36:3	0.015	±	0.001	0.014	±	0.001	0.2343
PC O-36:5	0.009	±	0.000	0.007	±	0.000	0.0073
PC O-38:0	0.005	±	0.001	0.002	±	0.000	0.0246
PC O-38:1	0.035	±	0.002	0.029	±	0.001	0.0157
PC O-40:1	0.076	±	0.004	0.073	±	0.002	0.6335
PC O-40:4	0.003	±	0.000	0.002	±	0.000	0.0223
PC O-40:6	0.003	±	0.000	0.002	±	0.000	0.0026
PC O-42:4	0.012	±	0.001	0.009	±	0.001	0.0399
PC O-44:4	0.013	±	0.002	0.011	±	0.001	0.3539
PE 34:1	0.130	±	0.007	0.183	±	0.009	0.0003
PE 34:2	0.008	±	0.001	0.013	±	0.001	0.0070
PE 36:1	0.111	±	0.007	0.160	±	0.010	0.0009
PE 36:2	0.217	±	0.009	0.291	±	0.016	0.0012
PE 36:3	0.012	±	0.001	0.023	±	0.002	0.0005
PE 36:4	0.068	±	0.004	0.087	±	0.004	0.0029
PE 38:1	0.012	±	0.001	0.020	±	0.002	0.0028
PE 38:2	0.032	±	0.002	0.040	±	0.002	0.0035
PE 38:4	0.381	±	0.026	0.527	±	0.031	0.0026
PE 38:5	0.130	±	0.006	0.150	±	0.008	0.0545
PE 38:6	0.285	±	0.023	0.388	±	0.025	0.0073
PE 40:4	0.014	±	0.002	0.023	±	0.003	0.0337
PE 40:6	0.880	±	0.080	1.317	±	0.101	0.0037
PE 40:7	0.067	±	0.004	0.091	±	0.009	0.0515
PE 44:10	0.012	±	0.001	0.012	±	0.001	0.9480
PE O-34:1	0.009	±	0.001	0.022	±	0.002	0.00001
PE O-34:2	0.196	±	0.010	0.272	±	0.015	0.0009
PE O-34:3	0.003	±	0.000	0.007	±	0.001	0.0068
PE O-36:2	0.220	±	0.015	0.342	±	0.025	0.0007
PE O-36:3	0.362	±	0.018	0.488	±	0.031	0.0028
PE O-36:4	0.024	±	0.002	0.036	±	0.004	0.0107
PE O-36:5	0.012	±	0.003	0.024	±	0.003	0.0132

PE O-38:2	0.061	±	0.004	0.098	±	0.008	0.0010
PE O-38:3	0.129	±	0.007	0.172	±	0.013	0.0093
PE O-38:4	0.026	±	0.001	0.040	±	0.004	0.0042
PE O-38:5	0.204	±	0.012	0.281	±	0.017	0.0021
PE O-38:6	0.127	±	0.005	0.147	±	0.009	0.0656
PE O-38:7	0.161	±	0.015	0.219	±	0.015	0.0129
PE O-40:5	0.061	±	0.003	0.087	±	0.005	0.0006
PE O-40:6	0.103	±	0.005	0.127	±	0.008	0.0251
PE O-40:7	0.423	±	0.043	0.625	±	0.049	0.0069
PE O-40:8	0.074	±	0.006	0.094	±	0.006	0.0348
PG 34:1	0.045	±	0.002	0.055	±	0.003	0.0250
PG 36:2	0.008	±	0.000	0.010	±	0.001	0.0479
PG 36:4	0.002	±	0.000	0.002	±	0.000	0.0379
PG 38:4	0.002	±	0.000	0.004	±	0.001	0.0661
PG 42:8	0.004	±	0.001	0.003	±	0.000	0.1725
PG 44:12	0.016	±	0.001	0.020	±	0.001	0.0813
PI 34:2	0.002	±	0.000	0.002	±	0.000	0.1074
PI 36:1	0.010	±	0.001	0.013	±	0.001	0.0057
PI 36:2	0.006	±	0.000	0.007	±	0.001	0.1940
PI 36:4	0.076	±	0.003	0.091	±	0.004	0.0180
PI 38:4	0.443	±	0.024	0.566	±	0.033	0.0077
PI 38:5	0.099	±	0.005	0.116	±	0.006	0.0613
PI 38:6	0.024	±	0.001	0.028	±	0.002	0.1005
PI 40:4	0.002	±	0.000	0.002	±	0.000	0.4137
PI 40:6	0.028	±	0.001	0.037	±	0.002	0.0038
PI 40:7	0.013	±	0.001	0.015	±	0.001	0.3191
PS 34:1	0.039	±	0.002	0.048	±	0.002	0.0017
PS 34:2	0.002	±	0.000	0.002	±	0.000	0.0096
PS 36:1	0.383	±	0.018	0.455	±	0.024	0.0271
PS 36:2	0.269	±	0.009	0.319	±	0.013	0.0052
PS 36:3	0.003	±	0.000	0.006	±	0.001	0.0150
PS 36:4	0.005	±	0.000	0.005	±	0.000	0.3847
PS 38:1	0.049	±	0.003	0.059	±	0.004	0.0417
PS 38:2	0.023	±	0.001	0.025	±	0.001	0.2347
PS 38:4	0.085	±	0.002	0.094	±	0.003	0.0131

PS 38:5	0.030	±	0.001	0.030	±	0.001	0.9908
PS 40:1	0.012	±	0.001	0.016	±	0.001	0.0121
PS 40:2	0.012	±	0.001	0.014	±	0.001	0.1651
PS 40:4	0.039	±	0.001	0.045	±	0.002	0.0085
PS 40:6	0.621	±	0.044	0.778	±	0.052	0.0346
PS 40:7	0.021	±	0.001	0.024	±	0.002	0.1953
PS 42:1	0.004	±	0.000	0.007	±	0.001	0.0033
PS 42:2	0.005	±	0.000	0.007	±	0.001	0.0010
PS 42:9	0.033	±	0.002	0.033	±	0.003	0.9588
PS 44:12	0.036	±	0.004	0.040	±	0.004	0.5527

2.2.4 Supplementary Table S3. Lipid species detected in WT and KO cortex

	Average mol% ± SEM						
	**WT**			**PCSK9 KO**			**p value**
FC	61.421	±	1.354	60.202	±	1.009	0.4810
DAG 32:0	0.011	±	0.004	0.010	±	0.001	0.7776
DAG 34:1	0.061	±	0.006	0.062	±	0.003	0.8732
DAG 34:2	0.006	±	0.003	0.004	±	0.001	0.4237
DAG 36:1	0.066	±	0.013	0.061	±	0.005	0.7103
DAG 36:2	0.023	±	0.003	0.023	±	0.001	0.9266
DAG 38:4	0.087	±	0.003	0.091	±	0.005	0.4891
Cer 34:1;2	0.017	±	0.001	0.018	±	0.001	0.6005
Cer 36:1;2	0.497	±	0.015	0.557	±	0.026	0.0572
Cer 36:2;2	0.175	±	0.004	0.187	±	0.007	0.1405
Cer 38:1;2	0.028	±	0.001	0.031	±	0.001	0.0896
Cer 42:1;2	0.002	±	0.001	0.003	±	0.001	0.1835
Cer 42:2;2	0.056	±	0.002	0.070	±	0.005	0.0318
GM3 34:1;2	0.002	±	0.001	0.004	±	0.001	0.1339
GM3 36:1;2	0.033	±	0.001	0.033	±	0.001	0.8474
GM3 42:2;2	0.013	±	0.001	0.009	±	0.001	0.0083
HexCer 36:1;2	0.055	±	0.005	0.051	±	0.002	0.4208
HexCer 42:1;2	0.196	±	0.008	0.247	±	0.010	0.0010
HexCer 42:2;2	0.650	±	0.022	0.774	±	0.038	0.0122
SHexCer 42:1;2	0.007	±	0.001	0.013	±	0.002	0.0193
SHexCer 42:2;2	0.079	±	0.008	0.096	±	0.008	0.1638
SM 34:1;2	0.020	±	0.001	0.019	±	0.001	0.6922
SM 36:1;2	0.717	±	0.014	0.749	±	0.027	0.2964
SM 36:2;2	0.059	±	0.003	0.057	±	0.002	0.5326
SM 40:1;2	0.007	±	0.001	0.008	±	0.001	0.3751
SM 42:2;2	0.085	±	0.003	0.097	±	0.004	0.0326
LPA 16:0	0.007	±	0.001	0.006	±	0.001	0.2162
LPA 18:0	0.013	±	0.001	0.008	±	0.001	0.0015
LPA 18:1	0.010	±	0.001	0.011	±	0.002	0.6973
LPC 16:0	0.097	±	0.003	0.096	±	0.004	0.7435
LPC 18:0	0.039	±	0.001	0.040	±	0.001	0.8352
LPC 18:1	0.027	±	0.001	0.026	±	0.001	0.8916

LPC 18:3	0.026	±	0.002	0.020	±	0.003	0.1553
LPC 20:3	0.006	±	0.001	0.008	±	0.001	0.3985
LPC 20:4	0.007	±	0.001	0.008	±	0.001	0.6501
LPE 16:0	0.021	±	0.001	0.018	±	0.001	0.0600
LPE 18:0	0.035	±	0.002	0.027	±	0.002	0.0185
LPE 18:1	0.070	±	0.003	0.069	±	0.003	0.7030
LPE 20:4	0.101	±	0.004	0.098	±	0.005	0.6620
LPE 22:4	0.049	±	0.002	0.048	±	0.002	0.6251
LPE 22:5	0.003	±	0.000	0.003	±	0.000	0.3208
LPE 22:6	0.225	±	0.007	0.220	±	0.011	0.7148
LPE O-16:1	0.007	±	0.000	0.005	±	0.000	0.0022
LPE O-18:1	0.008	±	0.001	0.006	±	0.000	0.0350
LPE O-18:2	0.006	±	0.001	0.003	±	0.000	0.0054
LPI 16:0	0.006	±	0.000	0.004	±	0.001	0.0158
LPI 18:0	0.029	±	0.002	0.025	±	0.001	0.0608
LPI 20:4	0.007	±	0.001	0.005	±	0.001	0.0785
LPI O-18:0	0.004	±	0.001	0.004	±	0.001	0.9318
LPS 18:0	0.082	±	0.007	0.055	±	0.005	0.0061
LPS 18:1	0.031	±	0.003	0.027	±	0.002	0.2348
LPS 20:4	0.004	±	0.000	0.003	±	0.000	0.1648
LPS 22:6	0.043	±	0.003	0.035	±	0.003	0.0636
PA 34:1	0.031	±	0.004	0.027	±	0.002	0.3054
PA 36:1	0.016	±	0.003	0.013	±	0.002	0.3831
PA 36:2	0.015	±	0.003	0.010	±	0.002	0.1024
PA 38:4	0.006	±	0.001	0.004	±	0.001	0.2078
PA 40:6	0.004	±	0.000	0.002	±	0.000	0.0665
PC 30:0	0.086	±	0.003	0.086	±	0.002	0.9258
PC 32:0	4.638	±	0.138	4.707	±	0.129	0.7199
PC 32:1	0.402	±	0.014	0.404	±	0.010	0.8811
PC 32:2	0.002	±	0.000	0.001	±	0.000	0.3187
PC 34:0	0.674	±	0.021	0.702	±	0.022	0.3605
PC 34:1	8.912	±	0.260	9.154	±	0.246	0.5081
PC 34:2	0.310	±	0.009	0.315	±	0.007	0.6054
PC 34:3	0.531	±	0.037	0.555	±	0.019	0.5726
PC 34:4	0.048	±	0.003	0.048	±	0.002	0.9303

PC 36:1	2.371	±	0.070	2.495	±	0.075	0.2423
PC 36:2	0.732	±	0.020	0.745	±	0.020	0.6613
PC 36:3	0.159	±	0.009	0.203	±	0.036	0.2572
PC 36:4	2.616	±	0.099	2.694	±	0.077	0.5390
PC 36:5	0.053	±	0.002	0.054	±	0.002	0.6620
PC 38:1	0.081	±	0.002	0.082	±	0.003	0.8474
PC 38:2	0.076	±	0.002	0.077	±	0.002	0.6670
PC 38:4	1.796	±	0.054	1.878	±	0.055	0.3035
PC 38:5	0.333	±	0.012	0.330	±	0.011	0.8304
PC 38:6	1.302	±	0.030	1.410	±	0.038	0.0410
PC 40:1	0.021	±	0.001	0.023	±	0.001	0.0536
PC 40:2	0.021	±	0.001	0.022	±	0.001	0.4931
PC 40:4	0.113	±	0.003	0.117	±	0.003	0.3940
PC 40:6	0.660	±	0.015	0.716	±	0.021	0.0483
PC 40:7	0.446	±	0.015	0.473	±	0.014	0.1938
PC 40:9	0.141	±	0.010	0.148	±	0.005	0.5534
PC 42:1	0.018	±	0.001	0.023	±	0.001	0.0012
PC 42:2	0.011	±	0.001	0.014	±	0.001	0.0012
PC 42:4	0.004	±	0.000	0.005	±	0.000	0.4654
PC 42:5	0.003	±	0.000	0.004	±	0.000	0.4082
PC 42:7	0.032	±	0.004	0.029	±	0.001	0.5735
PC 42:8	0.008	±	0.001	0.009	±	0.001	0.2176
PC 42:9	0.059	±	0.004	0.066	±	0.002	0.1411
PC 44:10	0.006	±	0.000	0.007	±	0.000	0.1838
PC O-32:0	0.042	±	0.002	0.043	±	0.001	0.8604
PC O-32:1	0.031	±	0.001	0.030	±	0.001	0.6298
PC O-34:0	0.013	±	0.001	0.013	±	0.001	0.7567
PC O-34:1	0.116	±	0.004	0.116	±	0.003	0.9932
PC O-34:2	0.013	±	0.001	0.013	±	0.001	0.7250
PC O-34:3	0.002	±	0.000	0.002	±	0.000	0.6995
PC O-36:1	0.006	±	0.000	0.005	±	0.000	0.0688
PC O-36:2	0.007	±	0.001	0.009	±	0.000	0.1134
PC O-36:3	0.003	±	0.001	0.004	±	0.000	0.2722
PC O-36:4	0.019	±	0.001	0.019	±	0.001	0.9995
PC O-38:1	0.009	±	0.001	0.010	±	0.001	0.3057

PC O-38:5	0.019	±	0.002	0.019	±	0.001	0.9226
PC O-40:1	0.014	±	0.001	0.019	±	0.001	0.0008
PC O-40:4	0.007	±	0.001	0.007	±	0.001	0.9150
PC O-40:6	0.007	±	0.001	0.007	±	0.001	0.8161
PC O-42:4	0.008	±	0.001	0.013	±	0.001	0.0026
PC O-42:6	0.048	±	0.003	0.057	±	0.003	0.0379
PC O-44:4	0.028	±	0.001	0.028	±	0.001	0.8159
PE 34:1	0.080	±	0.008	0.082	±	0.003	0.8265
PE 36:1	0.074	±	0.009	0.081	±	0.004	0.4671
PE 36:2	0.108	±	0.006	0.105	±	0.003	0.6841
PE 36:4	0.116	±	0.005	0.106	±	0.004	0.1599
PE 38:4	0.612	±	0.073	0.653	±	0.020	0.5974
PE 38:5	0.128	±	0.012	0.125	±	0.004	0.8115
PE 38:6	0.432	±	0.017	0.413	±	0.012	0.3825
PE 40:4	0.059	±	0.006	0.064	±	0.004	0.5164
PE 40:6	0.987	±	0.083	1.018	±	0.036	0.7333
PE 40:7	0.084	±	0.007	0.078	±	0.005	0.5256
PE O-34:2	0.080	±	0.008	0.085	±	0.005	0.6282
PE O-36:2	0.057	±	0.008	0.069	±	0.005	0.2288
PE O-36:3	0.134	±	0.016	0.153	±	0.006	0.2679
PE O-36:5	0.084	±	0.006	0.082	±	0.003	0.7683
PE O-38:4	0.006	±	0.001	0.006	±	0.001	0.6929
PE O-38:5	0.231	±	0.027	0.256	±	0.009	0.3867
PE O-38:6	0.176	±	0.008	0.168	±	0.005	0.3927
PE O-38:7	0.346	±	0.045	0.383	±	0.011	0.4345
PE O-40:5	0.072	±	0.011	0.088	±	0.006	0.2380
PE O-40:6	0.073	±	0.007	0.069	±	0.005	0.6496
PE O-40:7	0.385	±	0.052	0.444	±	0.018	0.2955
PE O-40:8	0.154	±	0.015	0.169	±	0.006	0.3465
PG 34:1	0.025	±	0.002	0.021	±	0.001	0.0957
PG 36:1	0.003	±	0.001	0.001	±	0.000	0.1498
PG 36:2	0.005	±	0.001	0.004	±	0.000	0.1916
PG 36:4	0.010	±	0.001	0.009	±	0.001	0.5376
PG 38:4	0.006	±	0.000	0.006	±	0.001	0.5482
PG 44:12	0.014	±	0.001	0.015	±	0.001	0.6448

PI 36:1	0.010	±	0.001	0.009	±	0.000	0.2621
PI 36:2	0.003	±	0.000	0.002	±	0.000	0.0101
PI 36:4	0.117	±	0.012	0.108	±	0.006	0.5239
PI 38:4	0.521	±	0.058	0.473	±	0.031	0.4739
PI 38:5	0.094	±	0.013	0.094	±	0.006	0.9738
PI 38:6	0.015	±	0.002	0.016	±	0.001	0.7076
PI 40:6	0.011	±	0.001	0.010	±	0.001	0.4607
PI 40:7	0.011	±	0.002	0.010	±	0.002	0.7503
PI 40:8	0.003	±	0.000	0.002	±	0.000	0.0896
PS 34:1	0.035	±	0.004	0.037	±	0.002	0.7065
PS 36:1	0.191	±	0.021	0.199	±	0.009	0.7468
PS 36:2	0.080	±	0.011	0.093	±	0.005	0.2871
PS 36:4	0.006	±	0.001	0.005	±	0.001	0.3391
PS 38:2	0.006	±	0.001	0.005	±	0.001	0.5216
PS 38:4	0.084	±	0.012	0.089	±	0.005	0.6984
PS 38:5	0.015	±	0.002	0.015	±	0.002	0.8246
PS 38:6	0.011	±	0.001	0.009	±	0.001	0.3308
PS 40:1	0.003	±	0.001	0.002	±	0.000	0.2522
PS 40:2	0.004	±	0.001	0.003	±	0.000	0.3577
PS 40:4	0.077	±	0.007	0.074	±	0.004	0.7070
PS 40:6	1.550	±	0.079	1.418	±	0.078	0.2505
PS 40:7	0.035	±	0.004	0.038	±	0.002	0.4384
PS 42:9	0.113	±	0.012	0.103	±	0.011	0.5641
PS 44:12	0.016	±	0.002	0.019	±	0.001	0.1933
